# Spatial pattern-driven interpretable model and biological correlates in brain glioblastoma-lymphoma differentiation

**DOI:** 10.1016/j.isci.2026.117281

**Published:** 2026-08-21

**Authors:** Yi Wang, Haohui Chen, Kaiyan Su, Jianrui Li, Tianliang Zhan, Lingyu Kong, Qiang Xu, Gaoping Liu, Xiaorui Su, Wei You, Jianguo Xia, Ji Zhang, Weizhong Tian, Wei Liao, Qiang Yue, Bing Zhang, Weihua Liao, Wei Xing, Dante Mantini, Guangming Lu, Zhiqiang Zhang, Multi-Centre Cooperative Clinical Study Group for “Lesion-Phenotype Brain Mapping in Brain Tumours”

**Affiliations:** 1 Department of Radiology, Jinling Clinical Medical College, Nanjing Medical University, Nanjing 210002, China; 2 Department of Radiology, The Affiliated Taizhou People’s Hospital of Nanjing Medical University, Taizhou School of Clinical Medicine, Nanjing Medical University, Taizhou 225300, China; 3 Department of Radiology, Jinling Clinical Medical College, Nanjing University of Chinese Medicine, Nanjing 210002, China; 4 Department of Radiology, Jinling Hospital, Affiliated Hospital of Medical School, Nanjing University, Nanjing 210002, China; 5 Department of Radiology, General Hospital of Center Theater of PLA, Wuhan 430070, China; 6 Department of Radiology, Xiangya Hospital, Central South University, Changsha 410008, China; 7 Department of Radiology, Nanjing Drum Tower Hospital, Affiliated Hospital of Medical School, Nanjing University, Nanjing 210008, China; 8 Department of Radiology, West China Hospital of Sichuan University, Chengdu 610041, China; 9 The Clinical Hospital of Chengdu Brain Science Institute, School of Life Science and Technology, University of Electronic Science and Technology of China, Chengdu 611731, China; 10 Department of Radiology, Third Affiliated Hospital of Soochow University, Changzhou 213003, China; 11 Movement Control and Neuroplasticity Research Group, Biomedical Sciences, KU Leuven, 3001 Leuven, Belgium

**Keywords:** glioblastoma, primary central nervous system lymphoma, tumor probabilistic maps, spatial radiomics, transcriptomics

## Abstract

Glioblastoma (GBM) and primary central nervous system lymphoma (PCNSL) often exhibit overlapping appearances on routine MRI, complicating pre-treatment diagnosis. In 1,109 patients from five centers, we constructed standard-space tumor probabilistic maps and derived atlas-anchored spatial features to augment conventional radiomics. The spatial radiomics classifier outperformed radiomics alone (external test area under the ROC curve [AUC], 0.98) with acceptable calibration and decision curve benefit, and SHapley Additive exPlanations (SHAP)-enabled anatomy-grounded interpretation. Aligning tumor localization with the Allen Human Brain Atlas and a normative functional connectome linked GBM-enriched territories to developmental-oncogenic programs and network hubness, whereas PCNSL-enriched territories showed immune-inflammatory/proliferative programs, and associations with network hubness did not survive spatial-autocorrelation correction. These results provide shareable reference maps and an interpretable, multicenter-generalizing tool for GBM-PCNSL differentiation, while offering biological context for diagnosis-specific location susceptibility.

## Highlights

• Tumor probabilistic maps quantify GBM-PCNSL localization beyond visual assessment
• Atlas-anchored spatial radiomics outperforms conventional radiomics across centers
• Normative transcriptomics and connectomics contextualize spatial correlates

## Graphical abstract

**Figure:**
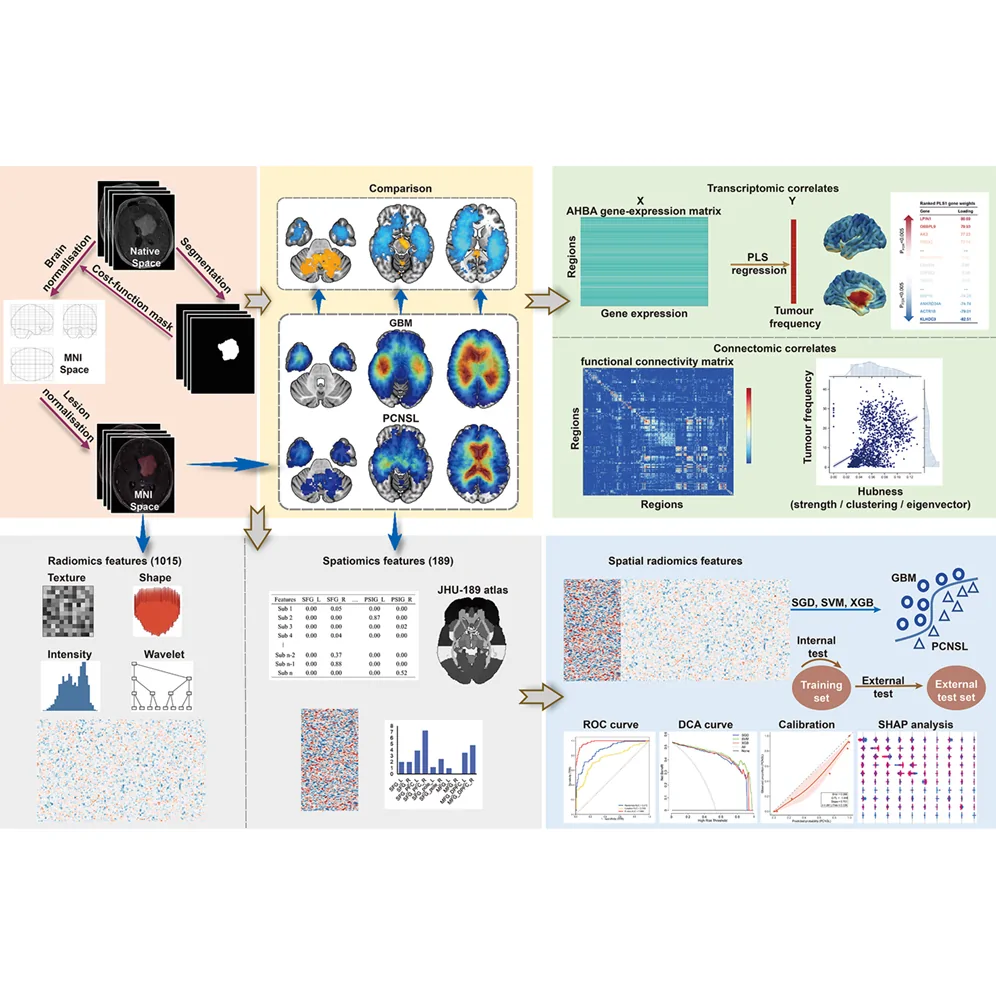

## Teaser

Neuroscience; Cancer; Transcriptomics; Machine learning

## Introduction

Glioblastoma (GBM) and primary central nervous system lymphoma (PCNSL) are highly malignant brain tumors.^1^ Despite distinct histopathology and treatment, they overlap clinically and radiologically, affecting older adults and presenting as deep, callosal “butterfly” lesions with avid enhancement and diffusion restriction on MRI.^2^^,^^3^^,^^4^ Differentiating GBM from PCNSL is a routine yet consequential diagnostic task.

Many quantitative imaging studies using MRI have addressed this task. Relative to GBM, PCNSL usually shows lower apparent diffusion coefficient on diffusion-weighted imaging,^5^^,^^6^ more prominent lipid and lactate peaks on MR spectroscopy,^7^^,^^8^ and typically lacks elevated perfusion because neovascularization is absent.^9^^,^^10^^,^^11^ Multimodal integration further improves performance.^12^^,^^13^ Radiomics, a high-throughput approach to quantitative image features,^14^ has been widely applied, with systematic reviews reporting high diagnostic accuracy.^15^^,^^16^

Most prior work, however, relies on region of interest (ROI) analyses that emphasize intralesional measurements while under-representing spatial location. Yet location carries clinical and biological meaning.^17^^,^^18^ PCNSL often tracks perivascular routes around deep medullary veins, producing perivascular “cuffing” and invasion of Virchow-Robin (VR) spaces and favoring deep periventricular and perivenular regions.^19^ In contrast, GBM relates to aberrant differentiation of neural stem/progenitor cells, conferring a propensity to arise near the subventricular zone (SVZ) and along glial lineage routes.^20^^,^^21^ Conventional location descriptors are visual and coarse. Voxel-based analyses in a common stereotactic space, originally developed for functional neuroimaging,^22^ are increasingly applied to focal lesions, including voxel-based lesion-symptom mapping (VLSM).^23^^,^^24^^,^^25^ By normalizing tumor masks to standard-space, these methods support statistical mapping, population-level tumor probabilistic maps, and formal testing of location-phenotype associations.^26^^,^^27^ Standard-space resources—Allen Human Brain Atlas (AHBA) transcriptomics and large-scale connectomes—permit normative correlational analyses that deepen pathophysiological understanding.^28^^,^^29^

Quantitative spatial descriptors can also operate as predictors. In earlier work, we intersected normalized lesion masks with a brain parcellation to obtain atlas-based spatial features and concatenated them with radiomics, achieving superior diagnostic accuracy, inter-site generalizability, and interpretability versus radiomics alone.^30^

In this multicenter MRI study, we first constructed whole-brain standard-space tumor probabilistic maps for GBM and PCNSL. We then trained and externally validated an integrated spatial radiomics classifier against conventional radiomics. Finally, we aligned the probabilistic maps with normative transcriptomic and functional connectomic datasets to relate location susceptibility to molecular programs and network hubness.

## Results

### Overview of the study

The final cohort included 1,109 patients from five centers, including 697 patients with GBM and 412 with PCNSL; demographic and clinical characteristics are summarized in Table 1, and patient selection and MRI acquisition details are provided in Figure S1 and Table S1.

**Table 1:** Demographic and clinical characteristics of the study cohort

Variables	GBM (*n* = 697)	PCNSL (*n* = 412)	*p* value
Age, years, median [IQR]	58 [49–66]	59 [50–67]	0.021^a^^,^^b^
Sex (male/female)	413/284	220/192	0.057^c^
Centers (1/2/3/4/5)	440/27/41/59/130	90/21/10/180/111	<0.001^b^^,^^c^
Cohort (development/external)	567/130	301/111	0.001^b^^,^^c^

Data are presented as median [IQR] or *n*.

GBM, glioblastoma; PCNSL, primary central nervous system lymphoma; IQR, interquartile range.

a Mann-Whitney *U* test.

b *p* < 0.05.

c Pearson’s χ^2^ test. All tests are two-sided.

### Tumor probabilistic maps

**Figure 1:**
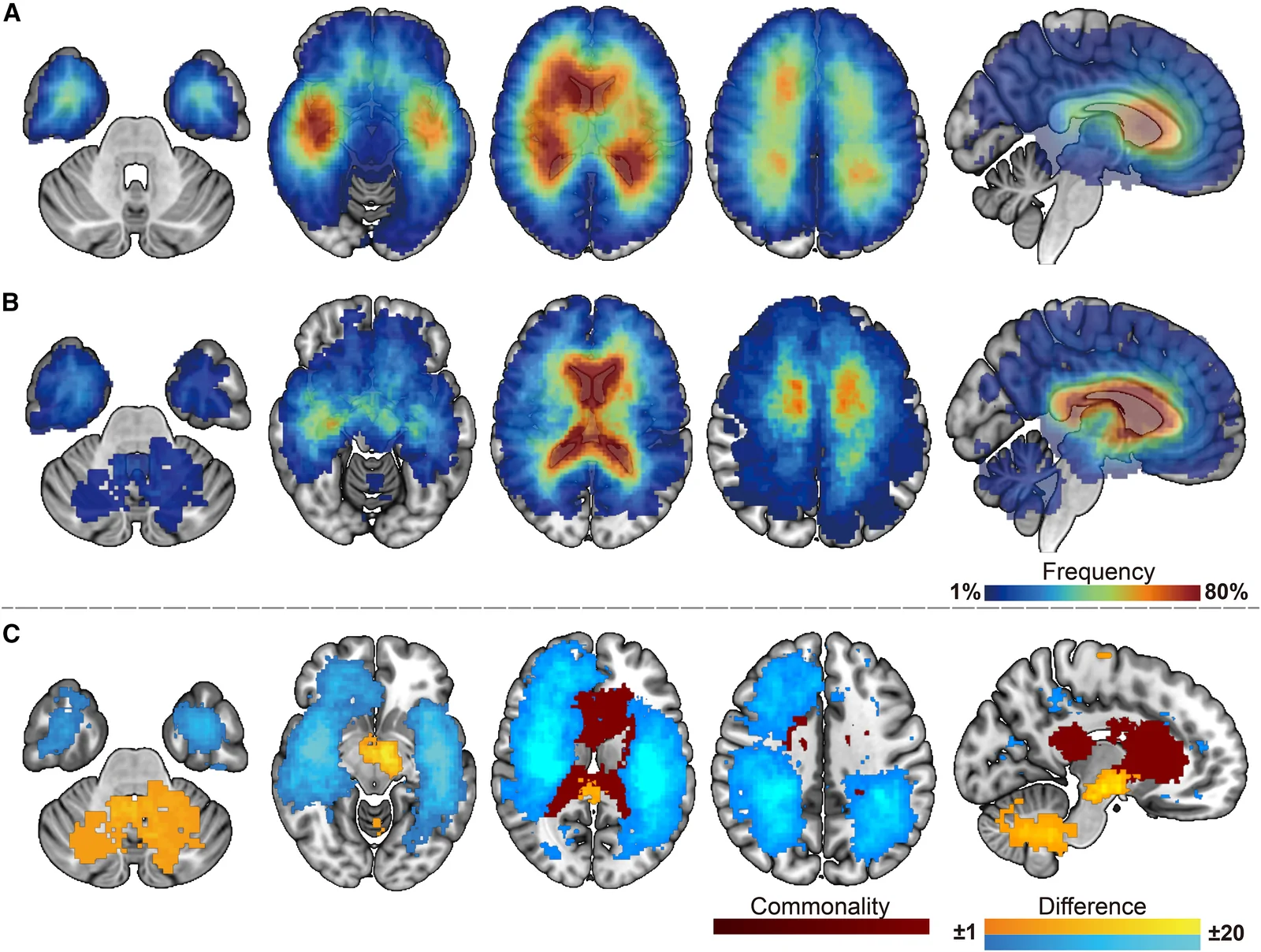
Probabilistic maps of tumor location (*N* = 1,109) (A) Voxel-wise frequency map for GBM (*n* = 697). (B) Voxel-wise frequency map for PCNSL (*n* = 412). Color bars indicate voxel-wise frequency (%). (C) Between-diagnosis map from voxel-wise χ^2^ with BH-FDR correction (q < 0.05): yellow, higher frequency in PCNSL; blue, higher frequency in GBM; red, common involvement (both ≥5%) without significant difference (q ≥ 0.05). See also Figure S1 and Tables S1, S2, and S3.

Voxel-wise tumor probabilistic maps showed a shared predilection of both GBM and PCNSL for the midline/periventricular deep white matter (Figures 1A and 1B). Between-diagnosis voxel-wise comparisons with false discovery rate (FDR) correction (q < 0.05) revealed clear divergences (Figure 1C; warm colors, PCNSL predilection; cool colors, GBM predilection): GBM more often involved the bilateral mesial temporal and insular regions (including hippocampal/entorhinal territories and the insula/external capsule) and the posterior temporal cortex, whereas PCNSL more frequently involved the brainstem (pons and midbrain), cerebellum (e.g., middle cerebellar peduncle), and major long tracts (e.g., corticospinal pathway). Occipital lobe involvement was low for both entities. All clusters reported here were identified after FDR control using 3D connected-component analysis with a 26-voxel neighborhood and a minimum extent of 100 voxels; peak coordinates, q values, and voxel counts are summarized in Table S2. Voxel-wise χ^2^ tests were performed in normalized space.

GBM had a larger median tumor volume than PCNSL (43.69 versus 16.81 mL), whereas multiple lesions were more frequent in PCNSL than in GBM (18.4% versus 10.3%). After adjustment for tumor volume, multifocality, center, age, and sex, 69 parcels remained significantly associated with diagnosis, including 68 with greater involvement in PCNSL and one with greater involvement in GBM (Table S3). In the single-lesion sensitivity analysis (*n* = 961), 51 parcels remained significant, of which 49 overlapped with the primary analysis. Diagnosis coefficients were strongly correlated between the primary and single-lesion analyses (Spearman ρ = 0.958).

### Model performance and interpretability

**Figure 2:**
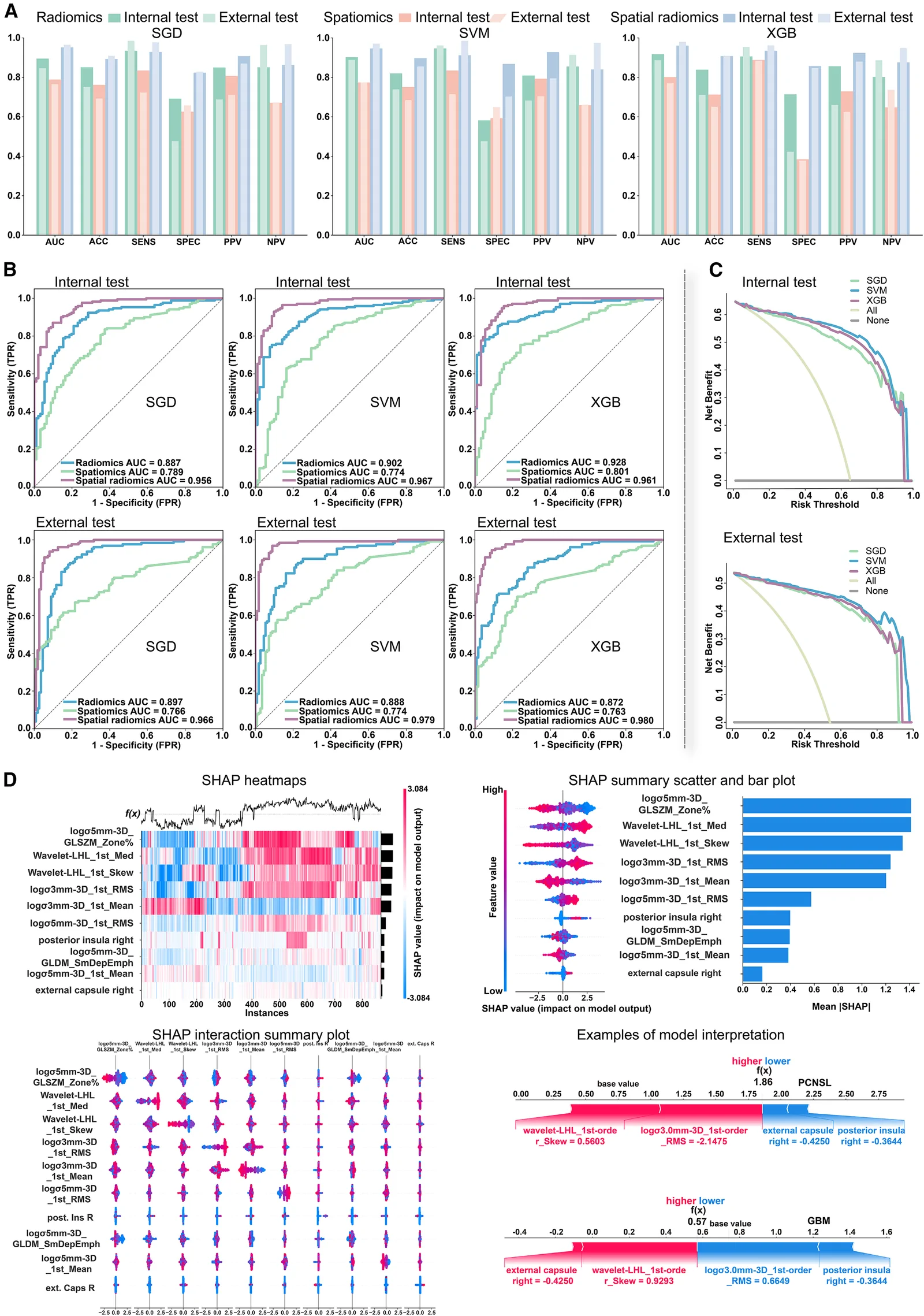
Model performance and SHAP interpretation (A) Summary metrics for three feature sets (radiomics, spatiomics, and spatial radiomics) across learners (SGD, SVM, and XGB) in the internal test cohort (*n* = 261) and the external test cohort (*n* = 241): area under the ROC curve (AUC), accuracy, sensitivity, specificity, and positive/negative predictive value (PPV/NPV). (B) ROC curves by learner for internal (top) and external (bottom) cohorts. (C) Decision curve analysis (net benefit vs. predicted GBM probability threshold) for spatial radiomics models in internal (top) and external (bottom) cohorts. (D) SHapley Additive exPlanations (SHAP) for the representative XGB spatial radiomics model: case-wise contribution heatmap, summary (“beeswarm”) plot, mean |SHAP| bar plot, interaction summary, and representative force plots. See also Figure S2 and Tables S4–S15.

Within the T1WI-CE-based modeling framework, the spatial radiomics model consistently outperformed radiomics and spatiomics across algorithms and cohorts (Table 2; Figures 2A and 2B). Internal and external area under the ROC curves (AUCs) were 0.956–0.967 and 0.966–0.980, respectively, with accuracies of 0.881–0.934 at the benchmarking threshold. Pairwise DeLong tests generally favored spatial radiomics after BH-FDR correction across pairwise contrasts (Table S4). Calibration was acceptable overall by Brier score (0.066–0.085; Table S5; Figure S2), but calibration-in-the-large and calibration slope showed deviations from ideal calibration in some external models, including a tendency toward external overprediction. Decision curve analysis, interpreted as preoperative triage based on the predicted probability of GBM, showed higher net benefit across thresholds (Figure 2C; Table S6).

**Table 2:** Performance of models for discriminating GBM from PCNSL

Dataset	Algorithm	Model	AUC (95% CI)	ACC	SENS	SPEC	PPV	NPV	F1 score
Internal test	SGD	Radiomics	0.887 (0.848, 0.926)	0.839	0.924	0.681	0.844	0.827	0.882
		Spatiomics	0.789 (0.735, 0.843)	0.762	0.835	0.626	0.807	0.671	0.821
		Spatial radiomics	0.956 (0.932, 0.979)	0.881	0.924	0.802	0.897	0.849	0.910
	SVM	Radiomics	0.902 (0.867, 0.938)	0.820	0.947	0.582	0.809	0.855	0.873
		Spatiomics	0.774 (0.718, 0.829)	0.751	0.835	0.593	0.793	0.659	0.814
		Spatial radiomics	0.967 (0.947, 0.987)	0.920	0.941	0.879	0.936	0.889	0.938
	XGB	Radiomics	0.928 (0.897, 0.958)	0.828	0.918	0.659	0.834	0.811	0.874
		Spatiomics	0.801 (0.748, 0.853)	0.724	0.882	0.429	0.743	0.661	0.806
		Spatial radiomics	0.961 (0.940, 0.983)	0.908	0.935	0.857	0.924	0.876	0.930
External test	SGD	Radiomics	0.897 (0.857, 0.937)	0.830	0.946	0.694	0.783	0.917	0.857
		Spatiomics	0.766 (0.707, 0.825)	0.693	0.723	0.658	0.712	0.670	0.718
		Spatial radiomics	0.966 (0.944, 0.989)	0.884	0.977	0.775	0.836	0.966	0.901
	SVM	Radiomics	0.888 (0.846, 0.930)	0.739	0.962	0.477	0.683	0.914	0.799
		Spatiomics	0.774 (0.716, 0.833)	0.685	0.715	0.649	0.705	0.661	0.710
		Spatial radiomics	0.979 (0.961, 0.997)	0.934	0.954	0.910	0.925	0.944	0.939
	XGB	Radiomics	0.872 (0.827, 0.917)	0.714	0.977	0.405	0.658	0.938	0.786
		Spatiomics	0.763 (0.703, 0.822)	0.622	0.846	0.360	0.608	0.667	0.707
		Spatial radiomics	0.980 (0.963, 0.997)	0.909	0.962	0.847	0.880	0.949	0.919

At the benchmarking probability threshold of 0.50, GBM was defined as the positive class and PCNSL as the negative class for threshold-based metrics. Therefore, SENS, PPV, and F1 refer to GBM classification, whereas SPEC and NPV refer to PCNSL as the negative class. AUC was calculated from continuous predicted probabilities.

GBM, glioblastoma; PCNSL, primary central nervous system lymphoma; SGD, stochastic gradient descent; SVM, support vector machine; XGB, extreme gradient boosting; AUC, area under the ROC curve; ACC, accuracy; SENS, sensitivity; SPEC, specificity; PPV, positive predictive value; NPV, negative predictive value; F1, F1 score; CI, confidence interval.

At the benchmarking probability threshold of 0.50, the spatial radiomics support vector machine (SVM) and extreme gradient boosting (XGB) models showed nearly identical external AUCs (0.979 and 0.980, respectively). XGB had slightly higher sensitivity than SVM (0.962 versus 0.954), whereas SVM had higher accuracy (0.934 versus 0.909), specificity (0.910 versus 0.847), positive predictive value (PPV) (0.925 versus 0.880), and F1 score (0.939 versus 0.919). Confusion matrices for the three spatial radiomics classifiers are provided in Table S7. XGB was used as the representative model for TreeSHAP interpretation (Figure 2D).

At the pre-specified operating point (τ = 0.30; positive class = GBM), the spatial radiomics XGB model achieved sensitivity 0.985 (95% confidence interval [CI], 0.95–1.00) and specificity 0.793 (0.71–0.86) in the external test set (*n* = 241), with PPV 0.848, negative predictive value (NPV) 0.978, and AUC 0.980 (Tables S8 and S9). At this high-sensitivity GBM operating point, a model-negative result, indicating low predicted GBM probability, would support consideration of PCNSL-oriented preoperative work-up and biopsy-first planning, including additional staging and cautious corticosteroid timing when clinically safe. Results were similar in the internal set (*n* = 261; sensitivity, 0.959; specificity, 0.824; AUC, 0.961). Radiomics and spatiomics ablations showed lower specificity at this operating point (Tables S8 and S9).

Generalizability was favorable for spatial radiomics, with external-internal differences of 0.010–0.019 for AUC and 0.001–0.014 for accuracy (Table S10). In center-stratified analyses, center-specific AUCs ranged from 0.958 to 1.000 for SGD, from 0.954 to 1.000 for SVM, and from 0.944 to 1.000 for XGB, with corresponding center-balanced macro-average AUCs of 0.968, 0.983, and 0.980, respectively (Tables S11 and S12). These findings indicate that the overall discriminatory performance was not driven by a single center. Calibration showed greater between-center variability than discrimination, particularly in small or imbalanced center-specific subsets, and was therefore interpreted cautiously.

In the interobserver robustness analysis of 50 paired segmentations, native-space tumor masks showed high agreement between the two readers, with a median Dice coefficient of 0.915 (IQR, 0.862–0.942) and a tumor-volume ICC of 0.967 (Table S13). After Montreal Neurological Institute (MNI) normalization, agreement remained acceptable, with a median Dice coefficient of 0.889 (IQR, 0.786–0.931) and a normalized tumor-volume ICC of 0.964. The atlas-anchored spatial features used in the modeling pipeline showed high reproducibility, with a median feature-level ICC of 0.966 (IQR, 0.911–0.986), and per-case spatial-feature vectors were highly concordant between readers (median cosine similarity, 0.988). In the ±2 mm registration-perturbation analysis, these spatial features remained stable, with a median feature-level ICC of 0.989 (IQR, 0.968–0.995), 92.4% of valid features showing ICC ≥ 0.90, and a median per-case cosine similarity of 0.990. These findings suggest that interobserver segmentation variability and small registration perturbations had a limited impact on the atlas-anchored spatial features used for model development.

Model hyperparameter search grids and the final selected configurations are provided in Table S14. SHapley Additive exPlanations (SHAP) for the representative spatial radiomics XGB identified texture families (e.g., GLSZM and wavelet first-order) and anatomical indicators (e.g., posterior insula and external capsule) that contributed to model predictions, yielding patient-level attributions (Figure 2D).

### Brain spatial transcriptomic correlates of tumor probabilistic maps

**Figure 3:**
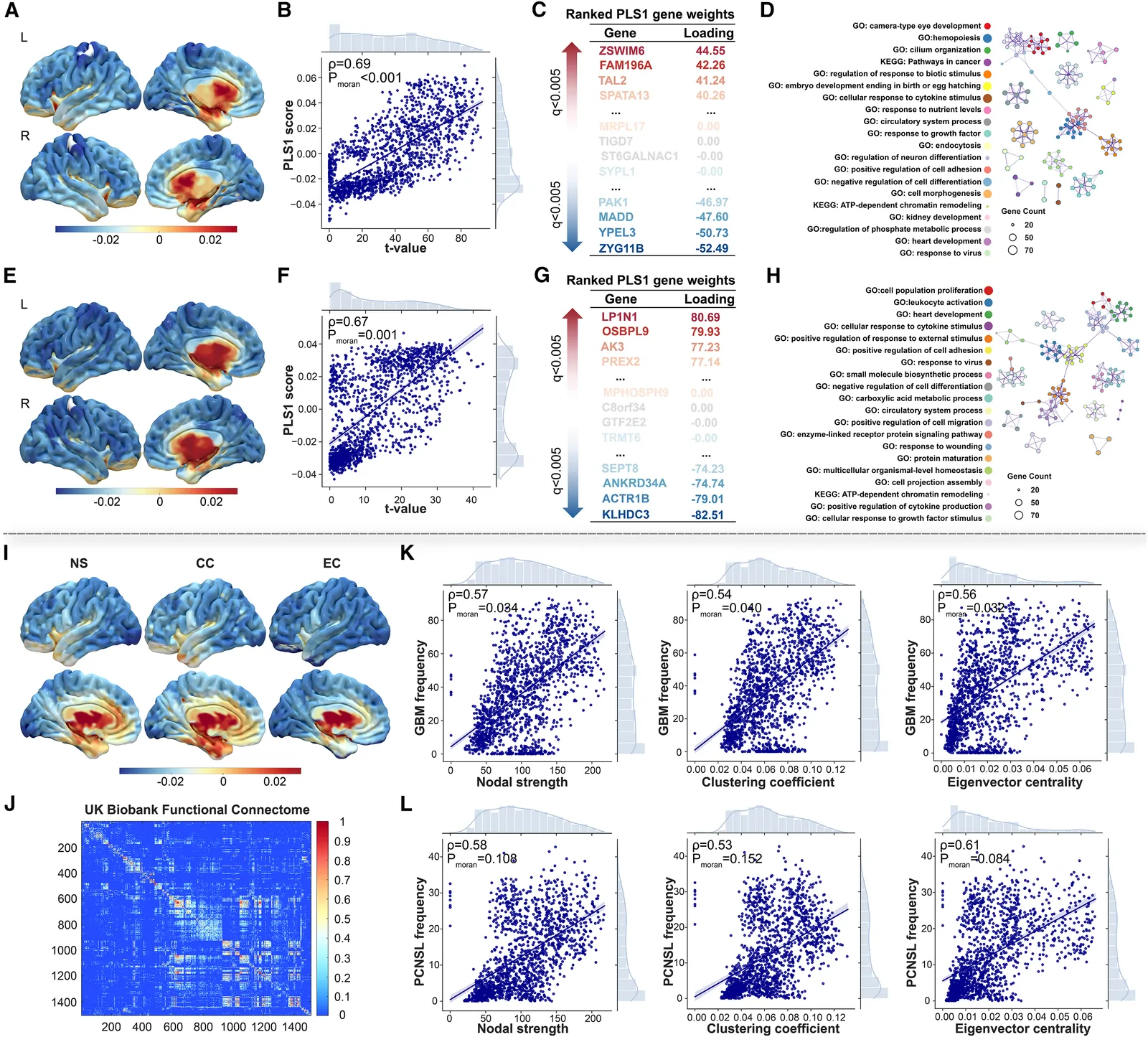
Spatial transcriptomic and connectomic contextualization (A–D) GBM. (A) Parcel-wise PLS1 score map. (B) Spatial correlation between PLS1 scores and parcel-wise GBM frequency (Spearman’s ρ; P_moran_ from Moran spectral randomization). (C) Ranked PLS1 gene weights with the FDR threshold indicated. (D) Gene Ontology (GO) and Kyoto Encyclopedia of Genes and Genomes (KEGG) enrichment network for positively weighted genes (q < 0.05). (E–H) PCNSL. (E) PLS1 score map. (F) Correlation between PLS1 scores and parcel-wise PCNSL frequency (Spearman’s ρ; P_moran_). (G) Ranked PLS1 gene weights with FDR-highlighted genes. (H) GO/KEGG enrichment for the positively weighted set (q < 0.05). (I–L) Connectome. (I) Parcel-wise hub metrics: nodal strength, weighted clustering coefficient, eigenvector centrality. (J) Parcel-by-parcel functional affinity matrix estimated from group independent component analysis (ICA, d = 100); nodes ordered by canonical networks. (K) GBM: correlations between tumor frequency and each hub metric (Spearman’s ρ; P_moran_). (L) PCNSL: analogous analyses; correlations not significant after Moran spectral randomization (P_moran_ > 0.05).

Partial least squares related brain-wide gene expression to regional tumor frequency for each entity. In GBM, PLS1 correlated with frequency (ρ = 0.69; Pmoran < 0.001) and was enriched for developmental/oncogenic and ciliogenesis-endocytosis programs (Gene Ontology/Kyoto Encyclopedia of Genes and Genomes [GO/KEGG] q < 0.05; Figures 3A–3D). In PCNSL, PLS1 correlated with frequency (ρ = 0.67; Pmoran = 0.001) and was enriched for immune/inflammatory and proliferative pathways (q < 0.05; Figures 3E–3H).

### Brain connectomic correlates of tumor probabilistic maps

Regional tumor frequency increased with hubness in the UK Biobank-derived functional connectome. For GBM, Spearman correlations with nodal strength (ρ = 0.57), weighted clustering coefficient (ρ = 0.54), and eigenvector centrality (ρ = 0.56) remained significant after Moran spectral randomization (Pmoran <0.05) and FDR control across the three metrics (q < 0.05; Figure 3K). For PCNSL, although uncorrected correlations were of similar magnitude (nodal strength ρ = 0.58; clustering coefficient ρ = 0.53; and eigenvector centrality ρ = 0.61), these associations did not survive Moran spectral randomization (all Pmoran >0.05 after FDR; Figure 3L).

## Discussion

Using a large multicenter MRI cohort of GBM and PCNSL, we constructed diagnosis-specific tumor probabilistic maps in standard space to quantify brain-wide spatial distribution patterns, revealing a shared predilection around the lateral ventricles and deep white matter alongside clear diagnosis-specific divergences. Importantly, these quantitative spatial descriptors enabled the development of differentiation models. A spatial radiomics model that fused atlas-based spatial features with conventional radiomics achieved superior diagnostic performance, stronger generalizability, and anatomy-based interpretability (with external validation). Anchored in a common stereotactic coordinate system, we further aligned tumor spatial patterns with normative transcriptomic and connectomic data, providing exploratory biological context for diagnosis-specific tumor topography.

Using automated voxel-based analysis, we generated population-level probabilistic maps that quantify the brain-wide spatial distributions of GBM and PCNSL. Both entities favored deep regions centered on the frontal lobes and rostral corpus callosum, consistent with established clinical knowledge.^2^^,^^3^^,^^4^ The occurrence of GBM is associated with the SVZ—a region enriched with neural stem and progenitor cells that may represent candidate cells of origin—as well as with anatomical patterns of glial-lineage differentiation and migration; it may also relate to the direction of GBM dissemination along white-matter tracts.^20^^,^^21^^,^^31^^,^^32^ By contrast, PCNSL is linked to VR perivascular spaces and deep medullary veins, where malignant B cell proliferation forms sleeve-like perivascular infiltration and VR-space invasion.^19^^,^^33^ These structures anatomically co-localize in the periventricular region surrounding the lateral ventricles. Moreover, the frontal/SVZ regions are characterized by higher immune tone and a relatively permissive BBB (blood-brain barrier), which might also be a plausible shared mechanism for co-predilection.^34^^,^^35^ We used the AHBA because it provides anatomically registered adult human brain gene-expression profiles, allowing regional tumor predilection to be interpreted in relation to normative transcriptional programs. Accordingly, our AHBA findings indicated that within the periventricular-rostral callosal-deep frontal overlap zone, upregulation of cilia biogenesis, endocytosis/growth-factor responses, neuronal differentiation, and nutrient signaling (“developmental-oncogenic” modules) was more consistent with GBM, whereas upregulation of leukocyte activation, cytokine responses/production, and adhesion/migration (“immune-inflammatory/proliferative” modules) characterized PCNSL-enriched territories.

Notably, the probabilistic maps also revealed distinct spatial distribution patterns between the two tumors: (1) compared with PCNSL, GBM was more enriched in the bilateral mesial temporal lobes and insular lobes. The medial temporal lobe (particularly the hippocampal dentate gyrus) represents another region enriched with neural stem/progenitor cells, aside from the subependymal zone.^36^^,^^37^ Our AHBA findings indicated that these regions were enriched for glioma-relevant programs centered on neuronal differentiation, ciliogenesis, and the RTK (receptor tyrosine kinase)-endocytosis axis, with comparatively weaker association to PCNSL-associated immune/leukocyte-activation gene sets, consistent with prior work.^38^ (2) In contrast to GBM, PCNSL showed a higher predilection for the brainstem (pons/midbrain) and cerebellum; this brainstem predilection may relate to the structure of deep medullary veins, and sentinel-like lymphoid lesions in some settings likewise suggest a brainstem affinity.^2^ Our AHBA analysis demonstrated that this brainstem region was dominated by pathways involved in immune effectors, endothelial receptor signaling, cytokine production/response, and adhesion/migration, while expression of development/progenitor cell-related markers was relatively low. (3) Although neither tumor was highly enriched in the cerebellum, a statistically significant relative difference was observed between them. GBM was rarely observed; this pattern may relate to regional differences in neural progenitor niches, local gene-expression programs, and microenvironmental context, whereas PCNSL showed relative alignment with an immune-vascular background.^19^^,^^35^^,^^39^ In our AHBA context, the cerebellum showed little prominence of developmental/progenitor and receptor-signaling-membrane-trafficking gene sets, but a higher baseline of immune effector, endothelial receptor, and adhesion/migration modules—features compatible with a VR perivascular niche—providing a possible context for the lower involvement of GBM and relatively higher involvement of PCNSL. (4) Both tumors were comparatively rare in the occipital lobe, plausibly reflecting lobe-specific histology and molecular context. GBM initiation depends on neural progenitor-like reservoirs, whereas PCNSL arises from malignant proliferation of mature B cells.^19^^,^^21^ Consistent with this, our AHBA analysis demonstrated that the occipital cortex shows a low baseline for both classes of transcriptional programs—developmental/oncogenic and immune-inflammatory. In addition, the occipital lobe lies relatively distant from the SVZ/subgranular zone (SGZ) neurogenic niches and is traversed by a sparser network of deep medullary veins and VR perivascular conduits,^40^ providing a plausible context for the low observed involvement of both GBM and PCNSL. The parcel-level sensitivity analyses supported the robustness of the principal diagnosis-related spatial pattern. Although GBM lesions were generally larger and multifocal disease was more frequent in PCNSL, the preferential involvement of periventricular, deep, brainstem, and infratentorial structures by PCNSL remained evident after adjustment for tumor volume, multifocality, center, age, and sex and after exclusion of multifocal cases. Nevertheless, residual differences in referral patterns and other unmeasured center-related factors cannot be completely excluded.

We further used a normative functional connectome because it captures population-level network organization and hubness, allowing tumor topography to be examined in relation to large-scale network architecture. Normative connectomic association analysis revealed a significant spatial coupling with network hubness in GBM, consistent with previous reports.^28^^,^^38^^,^^41^ According to the “hub vulnerability” hypothesis, brain disorders preferentially affect network hubs.^42^ More broadly, oncogenesis is more likely in hubs that carry heavy functional loads, incur high metabolic demands, concentrate short- and long-range fiber convergence, and maintain an active microenvironment.^28^ Intriguingly, this spatial coupling pattern was not confirmed for PCNSL after spatial-autocorrelation correction, although the uncorrected correlations were of similar magnitude, suggesting that “hub vulnerability” may not be a universal rule but rather exhibits disease specificity. We speculate that the divergence reflects distinct tumorigenic programs: neuroepithelial tumors such as GBM can be viewed as an aberrant re-initiation or hijacking of developmental programs that proceed along radial units and are driven by key gliogenic transcription factors (e.g., ASCL1), yielding anatomical phenotypes that closely parallel the spatiotemporal logic of normal neurogenesis.^20^^,^^21^ Within this framework, the propensity of GBM to arise in functional hubs is unsurprising—these regions not only shoulder intense information-integration and metabolic load but also lie adjacent to, or are enriched for, neural stem-cell and oligodendrocyte progenitor-cell niches, conferring elevated oncogenic susceptibility.^28^ By contrast, PCNSL is a lymphoid tumor and may not share the same “neurogenesis-hub” coupling; its spatial distribution may be more closely related to deep medullary veins, VR perivascular channels, and the immune microenvironment, although the present normative connectome analysis cannot exclude weaker or context-dependent network-topological associations.^19^^,^^33^

The modeling component was intentionally restricted to T1WI-CE, the most consistently available and standardizable sequence across centers, to isolate the incremental contribution of atlas-anchored tumor location. Accordingly, the model comparison demonstrates added value over T1WI-CE-derived radiomics and atlas-based spatial baselines. Traditional clinical radiological experience based on visual assessment has long recognized similar tumor location in GBM and PCNSL,^2^^,^^4^ but has not clearly delineated between-diagnosis differences in location, nor quantified similarities and differences in a statistical manner. In contrast, automated voxel-based analysis can identify in an unbiased and fine-grained manner distinct spatial distribution patterns. By anchoring each lesion in a standard stereotactic space, voxel-based analysis transforms location from a coarse descriptor into a quantitative feature suitable for diagnostic modeling. Within this T1WI-CE-based framework, spatial radiomics leveraged intrinsic spatial information and achieved superior diagnostic performance compared with conventional radiomics and atlas-based spatial baselines. Furthermore, traditional radiomics that rely on ROI analysis are susceptible to inter-institutional/acquisition-protocol/operator variability, which has led to persistent criticism of insufficient model generalization.^43^^,^^44^ Atlas-anchored stereotactic location features may be less sensitive to some acquisition-related variations and may therefore provide complementary information that supports cross-center transportability.

The slightly higher external AUCs observed for the spatial radiomics models may reflect both robust ranking performance and center-specific differences in case composition, tumor presentation, acquisition protocols, or referral patterns. The additional center-stratified and center-balanced analyses showed consistently high discrimination, suggesting that the pooled performance was not driven by one large or particularly favorable center. Nevertheless, calibration varied more substantially across centers than discrimination, indicating that absolute probability estimates may require local recalibration before prospective deployment, and residual center-related heterogeneity cannot be completely excluded.

Another criticism of traditional radiomics lies in the poor biological interpretability of its features, as high-level lesion features generated by computers and the decision process are frequently opaque to clinicians.^45^ In contrast, spatial topographic features represent intuitive, anatomy-grounded descriptors in which each regional feature possesses a clear, domain-native meaning. Our SHAP analysis indicated that spatial features contributed substantially to model predictions, supporting traceable explanation of the classifier. In addition, aligned with human brain spatial transcriptomics (e.g., the AHBA)—which registers transcriptomic profiles to standardized coordinates,^46^ our spatial radiomics registered tumor radiomic features at each anatomical location to the same standardized coordinate space. From the perspective of the emerging field of “spatial multi-omics,”^47^^,^^48^ our findings suggest that spatial radiomics may provide a complementary imaging-derived perspective within the spatial multi-omics methods array.

In sum, this study constructed population-level tumor probabilistic maps of GBM and PCNSL in standard space. It identified distribution differences between these two types of CNS malignancies that are not readily revealed by traditional visual radiological diagnosis and extracted quantitative location features for building diagnostic models. The spatial radiomics model showed improved diagnostic performance, favorable generalizability, and anatomy-grounded interpretability. The shared standard coordinate system further permitted integration of lesion spatial features with the human brain transcriptome and connectome, providing exploratory disease-specific biological context. This work provides a promising research framework for diagnostic brain imaging that may be extensible to other brain diseases and suggests that spatial radiomics may contribute to the spatial multi-omics methods array.

### Limitations of the study

Our study has several limitations. First, it used a retrospective design. Although external validation was completed and DCA suggested potential clinical value, the approach has yet to be evaluated prospectively in real-world settings. Second, model development was limited by several sources of incomplete information and residual heterogeneity. The modeling framework was based on T1WI-CE-derived features and did not include an expert-reader comparator or a full multiparametric MRI/clinical baseline model; therefore, the reported performance should be interpreted as incremental value over T1WI-CE-derived radiomics and spatial baselines. Incorporating multimodal MRI may further improve robustness and generalizability. In addition, IDH status was unavailable in 255 historical GBM cases, limiting complete retrospective WHO CNS5 reclassification and representing a potential source of diagnostic heterogeneity; detailed corticosteroid exposure before baseline MRI or biopsy was not consistently available and could not be incorporated as a covariate. Because the independent external center was unseen during training, its radiomics features were evaluated without center-specific ComBat adjustment, preserving strict test-set independence but potentially leaving residual center-related feature-distribution differences. Third, analyses of spatial transcriptomics and connectomes in healthy adults only represent population-level observational correlation, which cannot directly support causal inference regarding disease mechanisms; subsequent studies need to integrate tumor-specific spatial transcriptomics^49^ or patient-specific brain connectomic analysis^32^ to strengthen mechanistic evidence.

## Resource availability

### Lead contact

Further information and requests for resources should be directed to and will be fulfilled by the lead contact, Zhiqiang Zhang (zhangzq2001@126.com).

### Materials availability

This study did not generate new unique reagents.

### Data and code availability

• Data supporting the findings are provided within the article and its supplemental information. Raw center-specific MRI scans cannot be made publicly available because of ethics committee/IRB restrictions and patient privacy considerations. De-identified derived data generated in this study, including lesion masks, feature matrices, tumor probability maps, and model-output files, are available from the corresponding author upon reasonable request and completion of a data-use agreement. Publicly available group-level resources used for biological contextualization include the AHBA microarray dataset (https://human.brain-map.org/static/download) and the UK Biobank group-ICA resting-state dataset (access subject to UK Biobank terms; https://www.fmrib.ox.ac.uk/ukbiobank/).
• The Standard sPace Lesion-Symptom Mapping (SPLSM) pipeline (version 1.0 beta), developed by our team and openly available at https://github.com/JLhos-fmri/SPLSM_version1.0, was used for MNI normalization, generation of tumor probabilistic maps, voxel-wise q-statistics, and atlas-anchored spatial feature extraction. The JHU-189 atlas used to derive the 189 atlas-anchored spatial features is implemented within the Clinical Frequency Analysis Toolkit and is available at https://github.com/JLhos-fmri/ClinicalFrequencyAnalysisToolkit. Radiomics feature extraction used PyRadiomics v3.0.1 (https://pyradiomics.readthedocs.io/), batch harmonization used neuroHarmonize v2.4.5 (https://github.com/rpomponio/neuroHarmonize), and additional tools included SPM12 (https://www.fil.ion.ucl.ac.uk/spm/), abagen (https://github.com/rmarkello/abagen), and the Brain Connectivity Toolbox (https://sites.google.com/site/bctnet/).
• This study did not generate microarray or RNA-seq datasets, and no standalone software package was generated.
• Any additional information required to reanalyze the data reported in this paper is available from the lead contact upon reasonable request.

## Acknowledgments

We thank all participating centers and staff for their contributions to data collection and quality control. This work was supported by the Major Instrument Development Project of the 10.13039/501100001809National Natural Science Foundation of China (82127806), the 10.13039/501100012166National Key Research and Development Program of China (2018YFA0701703), and the Taizhou School of Clinical Medicine, Nanjing Medical University (TZKY20250209).

## Author contributions

Conceptualization, Z.Z., Y.W., H.C., and J.L.; methodology, Z.Z., Y.W., H.C., and K.S., with guidance from Q.X. and Wei Liao; software, Y.W., H.C., and K.S.; formal analysis, Y.W., H.C., and K.S.; data curation, J.L., H.C., Y.W., T.Z., L.K., G. Liu, X.S., and W.Y.; resources, J.X., J.Z., W.T., Q.Y., B.Z., Weihua Liao, W.X., D.M., G. Lu, and M.C.C.C.S.G.; funding acquisition, Z.Z. and G. Lu; writing – original draft, Y.W. and Z.Z.; supervision, Z.Z.; data verification, Y.W. and H.C. All authors contributed to writing – review and editing. J.L. verified the underlying data. Z.Z. verified the key processed datasets. Q.X. contributed to methodology guidance and could not approve the final version; all remaining authors approved the submitted version.

## Declaration of interests

The authors declare no competing interests.

## STAR★Methods

### Key resources table

**Table:** 

REAGENT or RESOURCE	SOURCE	IDENTIFIER
**Depositeddata**		

Allen Human Brain Atlas microarray dataset	Allen Institute for Brain Science	https://human.brain-map.org/static/download
UK Biobank group-ICA resting-state dataset	UK Biobank/FMRIB	https://www.fmrib.ox.ac.uk/ukbiobank/

**Software andalgorithms**		

SPLSM pipeline (Version 1.0 beta)	GitHub	https://github.com/JLhos-fmri/SPLSM_version1.0
ITK-SNAP	ITK-SNAP developers	http://www.itksnap.org/
SPM12	Wellcome Center for Human Neuroimaging	https://www.fil.ion.ucl.ac.uk/spm/
PyRadiomics (version = 3.0.1)	PyRadiomics developers	https://pyradiomics.readthedocs.io/
neuroHarmonize (version = 2.4.5)	GitHub	https://github.com/rpomponio/neuroHarmonize
Python software (version = 3.12.2; conda-forge build)	Python Software Foundation/conda-forge	https://www.python.org/; https://conda-forge.org/
Python package: scikit-learn (version = 1.7.1)	scikit-learn developers	https://scikit-learn.org/
Python package: XGBoost (version = 3.0.2)	XGBoost developers	https://xgboost.readthedocs.io/
Python package: SHAP (version = 0.48.0)	SHAP developers	https://shap.readthedocs.io/
abagen	GitHub	https://github.com/rmarkello/abagen
DPABI	DPABI developers	http://rfmri.org/dpabi
Brain Connectivity Toolbox	Brain Connectivity Toolbox developers	https://sites.google.com/site/bctnet/
Metascape	Metascape	https://metascape.org/
R software (version = 4.4.3)	R Foundation	https://www.r-project.org/

**Other**		

JHU-189 atlas/Clinical Frequency Analysis Toolkit	GitHub	https://github.com/JLhos-fmri/ClinicalFrequencyAnalysisToolkit

### Experimental model and study participant details

This multicentre, retrospective diagnostic study was approved by the institutional ethics committees of all participating hospitals (No. 2024DZKY-019-01), with a waiver of written informed consent, and was conducted in accordance with the Declaration of Helsinki. The study participants were consecutive adults with pathologically confirmed GBM or PCNSL who underwent preoperative MRI across five tertiary centers between July 2016 and June 2024. Detailed diagnostic criteria, eligibility criteria, and cohort allocation are described below.

### Method details

#### Diagnostic, inclusion, and exclusion criteria

Pathological diagnosis of the two tumor types followed the WHO classification in effect at the time of diagnosis (WHO 2016 for cases diagnosed before 2021; WHO CNS5 thereafter). GBM was defined as glioblastoma per WHO 2016 or adult-type IDH-wildtype glioblastoma per WHO CNS5. For pre-2021 GBM cases, original pathology reports and available molecular information were reviewed in relation to WHO CNS5 terminology. IDH status was documented in 442/697 GBM cases (63.4%); cases with documented IDH mutation were considered astrocytoma, IDH-mutant, CNS WHO grade 4 under WHO CNS5 and were not included in the GBM cohort. Historical GBM cases without documented IDH status were retained according to the original institutional pathology diagnosis and were not retrospectively reclassified as IDH-wildtype GBM. Pre-2021 WHO grade II/III diffuse gliomas were not retrospectively added to the GBM cohort solely based on molecular criteria. PCNSL was required to be confined to the central nervous system and to have no evidence of systemic lymphoma on available baseline staging, including systemic CT and/or FDG-PET/CT, with bone marrow, ophthalmological, CSF, and HIV evaluation reviewed when available or clinically indicated.

Inclusion criteria were: (i) newly diagnosed, treatment-naïve GBM or PCNSL; and (ii) availability of preoperative contrast-enhanced T1-weighted imaging (T1WI-CE), with co-registered T2-weighted imaging (T2WI) available for anatomical reference and assessment of peritumoural oedema; both sequences were acquired within 14 days before biopsy or surgical resection. Exclusion criteria were: (i) prior cranial surgery, radiotherapy, or chemotherapy; (ii) recurrent tumor or secondary CNS lymphoma; and (iii) severe motion or metal artifacts, or incomplete brain coverage on MRI. Preoperative MRI was performed within 14 days prior to biopsy/surgery; no tumour-directed therapy was administered between imaging and tissue sampling.

#### Data acquisition and dataset description

From July 2016 to June 2024, consecutive adults with histopathologically confirmed GBM or PCNSL who underwent preoperative MRI were screened across five centers. Data were retrospectively retrieved from institutional PACS/clinical databases. In total, 1,109 patients were included (GBM, *n* = 697; PCNSL, *n* = 412; 633 men, 476 women; mean age, 56.8 ± 13.2 years). Sex was recorded from clinical records as male or female; gender identity was not available in the retrospective dataset. The reference standard was histopathology from surgical resection or stereotactic biopsy. Participating centers and the patient-selection flow diagram, including screened cases, exclusion reasons, final diagnostic counts, and centre-wise allocation, are shown in Figure S1. Demographics and clinical characteristics are summarised in Table 1.

Post-contrast T1-weighted MRI (T1WI-CE) was acquired on 11 scanners (1.5 T/3.0 T) from five vendors according to routine clinical protocols, including both 2D and 3D acquisitions; acquisition parameters and manufacturers are listed in Table S1. Exact post-contrast timing was not uniformly available in the retrospective metadata. Cases were included when whole-brain coverage and image quality were sufficient for tumor segmentation and spatial normalisation. All eligible cases within the accrual window were included.

#### Tumor segmentation, spatial normalisation, and population-level tumor probabilistic maps

Tumors were manually segmented on T1WI-CE in ITK-SNAP^50^ by a neuroradiologist and independently confirmed by a senior neuroradiologist; both were blinded to diagnosis and clinical information. Segmentation targeted the enhancing tumor on T1WI-CE; T2WI was reviewed only as an anatomical reference and for assessment of peritumoural oedema, and oedema was not segmented as an independent model input. For patients with multiple lesions, all visible enhancing lesions were included in a single patient-level tumor mask. Multifocality was defined as the presence of two or more spatially discontinuous enhancing lesions on the original MRI. Images were normalised to Montreal Neurological Institute (MNI) space using SPM12 with cost-function masking. After cost-function masking, two neuroradiologists independently performed visual quality control of MNI normalisation outside the lesion mask, assessing alignment of the midline (falx/AC–PC line), deep nuclei (caudate/putamen/thalamus), and ventricular margins against the MNI template. When an initial normalisation was judged suboptimal, normalisation was repeated with adjusted cost-function masks and default SPM12 settings. All scans (1,109/1,109; 100%) met the predefined QC criteria after repeat normalisation, where needed; no cases were excluded for misregistration. Voxel-wise tumor frequencies were computed to generate diagnosis-specific tumor probabilistic maps. Between-diagnosis differences were tested using voxel-wise χ^2^ tests in normalised space with Benjamini–Hochberg false discovery rate (FDR) control (q < 0.05).

To quantify segmentation and registration robustness, we performed an additional analysis in 50 paired cases. A second neuroradiologist independently segmented the enhancing tumor on native T1WI-CE, and agreement with the original segmentation was assessed using Dice coefficient and tumour-volume ICC. The paired masks were then processed using the same MNI-normalisation and JHU-189 atlas-extraction procedures as in the main analysis to evaluate the reproducibility of the atlas-anchored spatial features used in the modeling pipeline. Feature-level ICCs and per-case spatial-vector similarities were calculated between readers. To assess registration sensitivity, the Reader 1 MNI-normalised masks were shifted by ±2 mm along each of the three image axes, followed by re-extraction of the same atlas-anchored spatial features. Feature-level ICCs and per-case spatial-vector similarities were calculated between the original and perturbed masks.

To assess potential confounding of the spatial maps, additional parcel-level analyses were performed using the JHU-189 atlas,^51^ which contains 189 annotated regions (110 grey-matter, 68 white-matter, and 11 ventricular parcels). Regional involvement was defined as a parcel-wise lesion overlap greater than zero. For each parcel, logistic regression was performed with diagnosis as the primary independent variable, adjusting for log-transformed total tumor volume, multifocality, center, age, and sex; center was entered as a categorical fixed effect. Diagnosis-related *p* values were corrected across the 189 parcels using the Benjamini–Hochberg procedure, with q < 0.05 considered significant. The analysis was repeated after excluding all multifocal cases, with adjustment for tumor volume, center, age, and sex.

#### Radiomics and spatial feature extraction

All model-development features were derived from T1WI-CE images and T1WI-CE enhancing-tumour masks. T1WI-CE-derived radiomics features (shape, first-order, texture, LoG-filtered, and wavelet-derived features) were extracted using Image Biomarker Standardisation Initiative (IBSI)-compliant PyRadiomics v3.0.1.^52^ Before radiomics extraction, T1WI-CE images were resampled to isotropic 1-mm spacing using B-spline interpolation. Image intensities were normalised using the PyRadiomics normalisation option with a normalisation scale of 100 and a voxel-array shift of 300. Grey-level discretisation was performed using a fixed bin width of 25, and LoG sigma values were set to 3.0 and 5.0. No skull stripping or separate bias-field correction was performed before feature extraction because radiomics features were extracted within the enhancing-tumour mask. In total, 1,015 features were obtained. NeuroCombat harmonisation was implemented using neuroHarmonize v2.4.5.^53^ Harmonisation parameters were estimated using only the training partition, with imaging center as the batch variable and age and sex as covariates. Neither held-out test set was used to estimate or update harmonisation parameters. Because the external center was not represented during training, no centre-specific ComBat adjustment was applied to its radiomics features; they were processed using the locked downstream pipeline.

After normalisation to MNI space, atlas-anchored spatial features were extracted using the JHU-189 atlas described above. For each subject, parcel-wise atlas-anchored lesion-involvement descriptors were computed to form a subject-by-region spatial feature matrix.

#### Machine learning–based differentiation model development

Cases were split by center: one held-out center formed the external test set (*n* = 241); the remaining cases (*n* = 868) were divided 7:3 into training (*n* = 607) and internal test (*n* = 261), stratified by site and diagnosis. Three supervised learners—stochastic gradient descent (SGD; log loss), support vector machine (RBF), and extreme gradient boosting (XGB)—were trained on three predefined feature sets: radiomics, defined as T1WI-CE-derived conventional radiomics features; spatiomics, defined as atlas-anchored spatial descriptors derived from standard-space lesion location; and spatial radiomics, defined as the fusion of radiomics and spatiomics features.

Variance filtering, standardisation, correlation pruning (|Spearman ρ| > 0.90), feature reduction, and model fitting were restricted to the training data. Hyperparameters were selected by stratified 5-fold cross-validation using AUC (Table S14). The selected pipeline was then fitted on the complete training partition, and the held-out internal and external test sets were used exclusively for final performance evaluation, without preprocessing estimation, feature selection, hyperparameter tuning, recalibration, or threshold optimisation (Table S15). Analysts were blinded to test outcomes until final evaluation. Analyses used a complete-case approach after predefined eligibility and image-quality exclusions (Figure S1); no imputation was performed.

#### Brain spatial transcriptomic correlates of tumor probabilistic maps

To relate tumor localisation to normative gene expression, parcel-wise tumor frequencies were aligned to the Allen Human Brain Atlas (AHBA). Following prior work,^28^^,^^38^^,^^41^ donor microarray data were processed with abagen^46^ per best-practice workflows^54^ and assigned in MNI space to the JHU-189 parcellation uniformly subdivided to 1,512 micro-parcels (JHU-1512). Region-wise expression (homotopic mirroring and donor averaging) was filtered by differential stability (DS > 0.10), yielding a 1,512 × 15,633 matrix. For each diagnosis, partial least squares (PLS) related regional tumor frequency (z-scored) to expression; association strength was summarised by Spearman’s ρ and tested against Moran spectral randomisation (5,000 surrogates) to control spatial autocorrelation.^55^ Gene weights on PLS1 were bootstrapped (5,000), converted to z-scores and FDR-thresholded (q < 0.05) to define positively weighted genes for GO/KEGG enrichment (Metascape), using the abagen-filtered background.

#### Brain connectomic correlates of tumor probabilistic maps

To test coupling with functional network topology, we derived a parcel-level affinity network from UK Biobank group-independent component analysis (ICA) spatial maps (d = 100).^56^ ICA maps were resampled to the 1,512-parcel grid with DPABI^57^; inter-parcel affinity was the Pearson correlation of loading vectors, with negative edges set to zero. On this weighted graph we computed nodal strength, weighted clustering coefficient and eigenvector centrality (Brain Connectivity Toolbox).^58^ Regional tumor frequency was correlated (Spearman) with each metric; significance used Moran spectral randomisation with Benjamini–Hochberg FDR across metrics.

### Quantification and statistical analysis

#### Statistical analyses for cohort, spatial, and biological-context analyses

Demographic differences between GBM and PCNSL were assessed using a two-sided Mann–Whitney U test for age and Pearson’s χ^2^ tests for categorical variables; in Table 1, ∗ denotes *p* < 0.05. Voxel-wise diagnosis differences were evaluated using χ^2^ tests with Benjamini–Hochberg false discovery rate (FDR) correction. Parcel-level diagnosis associations were assessed using multivariable logistic regression adjusted for log-transformed total tumor volume, multifocality, center, age, and sex, with BH–FDR correction across 189 parcels; the single-lesion sensitivity analysis excluded multifocal cases. Interobserver and registration-perturbation robustness was quantified using Dice coefficients, intraclass correlation coefficients, cosine similarity, and binary parcel-involvement agreement. Transcriptomic and connectomic spatial associations were summarised using Spearman’s ρ and tested using Moran spectral randomisation with 5,000 surrogates; PLS1 gene weights were bootstrapped 5,000 times and FDR-corrected, and connectomic tests were FDR-corrected across the three hub metrics. All tests were two-sided unless otherwise specified.

#### Statistical analysis for model estimation

Model performance was summarised by the area under the receiver operating characteristic curve (AUC; 95% confidence interval [CI]). We also report accuracy, sensitivity, specificity, PPV, NPV and F1. AUCs were compared across feature sets (radiomics, spatiomics, spatial radiomics) and learners (SGD, SVM, XGB) using DeLong’s test on paired predictions. Generalisability was quantified as ΔAUC = AUC_external − AUC_internal (and ΔACC analogously). Clinical utility was assessed using decision curve analysis (DCA) across a range of threshold probabilities, with representative thresholds of 0.25 and 0.75 highlighted. For clinical interpretation, DCA was framed as preoperative triage based on the predicted probability of GBM: a model-positive result at a given GBM probability threshold was interpreted as supporting GBM-oriented surgical planning, whereas a low predicted GBM probability could prompt consideration of a PCNSL-oriented diagnostic pathway. Calibration used Brier score, calibration-in-the-large/slope and bootstrap reliability curves. Two-sided α = 0.05 with Benjamini–Hochberg FDR control. Reporting adhered to STARD/TRIPOD and aligned with CLAIM. Splits were training (*n* = 607), internal test (*n* = 261) and external test (*n* = 241). At the benchmarking probability threshold of 0.50, GBM was defined as the positive class and PCNSL as the negative class for the calculation of sensitivity, specificity, PPV, NPV, and F1 score. For the separate high-sensitivity analysis, GBM was also defined as the positive class, its predicted probability was used, and the operating threshold was set at τ = 0.30. The three spatial radiomics classifiers were evaluated as parallel algorithmic implementations. XGB was used as the representative model for TreeSHAP interpretation because of the native compatibility of TreeExplainer with tree-based predictions. Training-set performance is omitted to avoid optimism bias. To further assess center effects and external generalisability, we performed centre-stratified analyses using predictions from the locked spatial radiomics models. Centers 1–4 were evaluated using their respective patients in the held-out internal test set, whereas Center 5 comprised the independent external test set. Centre-balanced macro-average AUC, accuracy, and Brier score were calculated as unweighted means of the centre-specific estimates. Centre-specific calibration estimates were interpreted cautiously when sample size or class composition limited reliable estimation. Analyses used Python 3.12.2 (scikit-learn 1.7.1; XGBoost 3.0.2; SHAP 0.48.0; neuroHarmonize 2.4.5) and R 4.4.3. Unless otherwise specified, n denotes the number of individual patients, and center denotes the participating tertiary hospital/site. Continuous variables are summarised as mean ± SD or median [IQR], as appropriate, and uncertainty is reported using 95% CIs where applicable; analysis-specific tests and sample sizes are provided in the Results, figure/table legends, and supplementary tables.

## Supplemental information

Document S1. Figures S1 and S2; Tables S1, S2, and S4–S15

Table S3. Parcel-level diagnosis associations in the primary adjusted and single-lesion sensitivity analyses, related to STAR Methods

## References

[1] Price M., Ballard C., Benedetti J., Neff C., Cioffi G., Waite K.A., Kruchko C., Barnholtz-Sloan J.S., Ostrom Q.T. (2024). CBTRUS Statistical Report: Primary Brain and Other Central Nervous System Tumors Diagnosed in the United States in 2017-2021. Neuro Oncol..

[2] Pons-Escoda A., Naval-Baudin P., Velasco R., Vidal N., Majós C. (2023). Imaging of Lymphomas Involving the CNS: An Update-Review of the Full Spectrum of Disease with an Emphasis on the World Health Organization Classifications of CNS Tumors 2021 and Hematolymphoid Tumors 2022. AJNR. Am. J. Neuroradiol..

[3] Vollmuth P., Karschnia P., Sahm F., Park Y.W., Ahn S.S., Jain R. (2025). A Radiologist's Guide to IDH-Wildtype Glioblastoma for Efficient Communication With Clinicians: Part I-Essential Information on Preoperative and Immediate Postoperative Imaging. Korean J. Radiol..

[4] Bjorland L.S., Dæhli Kurz K., Fluge Ø., Gilje B., Mahesparan R., Sætran H., Ushakova A., Farbu E. (2022). Butterfly glioblastoma: Clinical characteristics, treatment strategies and outcomes in a population-based cohort. Neurooncol. Adv..

[5] Kamimura K., Nakano T., Hasegawa T., Nakajo M., Yamada C., Kamimura Y., Akune K., Ejima F., Ayukawa T., Nagano H. (2023). Differentiating primary central nervous system lymphoma from glioblastoma by time-dependent diffusion using oscillating gradient. Cancer Imaging.

[6] Romano A., Palizzi S., Romano A., Moltoni G., Di Napoli A., Maccioni F., Bozzao A. (2023). Diffusion Weighted Imaging in Neuro-Oncology: Diagnosis, Post-Treatment Changes, and Advanced Sequences-An Updated Review. Cancers (Basel).

[7] Yamasaki F., Takayasu T., Nosaka R., Amatya V.J., Doskaliyev A., Akiyama Y., Tominaga A., Takeshima Y., Sugiyama K., Kurisu K. (2015). Magnetic resonance spectroscopy detection of high lipid levels in intraaxial tumors without central necrosis: a characteristic of malignant lymphoma. J. Neurosurg..

[8] Lu S.S., Kim S.J., Kim H.S., Choi C.G., Lim Y.M., Kim E.J., Kim D.Y., Cho S.H. (2014). Utility of proton MR spectroscopy for differentiating typical and atypical primary central nervous system lymphomas from tumefactive demyelinating lesions. AJNR. Am. J. Neuroradiol..

[9] Pons-Escoda A., García-Ruíz A., Naval-Baudin P., Grussu F., Viveros M., Vidal N., Bruna J., Plans G., Cos M., Perez-Lopez R., Majós C. (2022). Diffuse Large B-Cell Epstein-Barr Virus-Positive Primary CNS Lymphoma in Non-AIDS Patients: High Diagnostic Accuracy of DSC Perfusion Metrics. AJNR. Am. J. Neuroradiol..

[10] Soydan H., Sözmen Cılız D., Cesur T., Tezgör Aksakal E. (2024). Primary brain lymphoma and glioblastoma: evaluation of DCE T1 and DSC T2 MRI perfusion findings. Acta Radiol..

[11] Suh C.H., Kim H.S., Jung S.C., Park J.E., Choi C.G., Kim S.J. (2019). MRI as a diagnostic biomarker for differentiating primary central nervous system lymphoma from glioblastoma: A systematic review and meta-analysis. J. Magn. Reson. Imag..

[12] Yu X., Hong W., Ye M., Lai M., Shi C., Li L., Ye K., Xu J., Ai R., Shan C. (2023). Atypical primary central nervous system lymphoma and glioblastoma: multiparametric differentiation based on non-enhancing volume, apparent diffusion coefficient, and arterial spin labeling. Eur. Radiol..

[13] Hung N.D., Anh N.N., Minh N.D., Huyen D.K., Duc N.M. (2023). Differentiation of glioblastoma and primary central nervous system lymphomas using multiparametric diffusion and perfusion magnetic resonance imaging. Biomed. Rep..

[14] Mayerhoefer M.E., Materka A., Langs G., Häggström I., Szczypiński P., Gibbs P., Cook G. (2020). Introduction to Radiomics. J. Nucl. Med..

[15] Guha A., Halder S., Shinde S.H., Gawde J., Munnolli S., Talole S., Goda J.S. (2024). How does deep learning/machine learning perform in comparison to radiologists in distinguishing glioblastomas (or grade IV astrocytomas) from primary CNS lymphomas?: a meta-analysis and systematic review. Clin. Radiol..

[16] Cassinelli Petersen G.I., Shatalov J., Verma T., Brim W.R., Subramanian H., Brackett A., Bahar R.C., Merkaj S., Zeevi T., Staib L.H. (2022). Machine Learning in Differentiating Gliomas from Primary CNS Lymphomas: A Systematic Review, Reporting Quality, and Risk of Bias Assessment. AJNR. Am. J. Neuroradiol..

[17] Luna L.P., Sherbaf F.G., Sair H.I., Mukherjee D., Oliveira I.B., Köhler C.A. (2021). Can Preoperative Mapping with Functional MRI Reduce Morbidity in Brain Tumor Resection? A Systematic Review and Meta-Analysis of 68 Observational Studies. Radiology.

[18] Gerritsen J.K.W., Mekary R.A., Pisică D., Zwarthoed R.H., Kilgallon J.L., Nawabi N.L., Jessurun C.A.C., Versyck G., Moussa A., Bouhaddou H. (2024). Onco-functional outcome after resection for eloquent glioblastoma (OFO): A propensity-score matched analysis of an international, multicentre, cohort study. Eur. J. Cancer.

[19] Ferreri A.J.M., Calimeri T., Cwynarski K., Dietrich J., Grommes C., Hoang-Xuan K., Hu L.S., Illerhaus G., Nayak L., Ponzoni M., Batchelor T.T. (2023). Primary central nervous system lymphoma. Nat. Rev. Dis. Primers.

[20] Akeret K., Weller M., Krayenbühl N. (2023). The anatomy of neuroepithelial tumours. Brain.

[21] Maher E.A., Furnari F.B., Bachoo R.M., Rowitch D.H., Louis D.N., Cavenee W.K., DePinho R.A. (2001). Malignant glioma: genetics and biology of a grave matter. Genes Dev..

[22] Friston K.J., Holmes A.P., Worsley K.J., Poline J.-P., Frith C.D., Frackowiak R.S.J. (1994). Statistical parametric maps in functional imaging: A general linear approach. Hum. Brain Mapp..

[23] Bates E., Wilson S.M., Saygin A.P., Dick F., Sereno M.I., Knight R.T., Dronkers N.F. (2003). Voxel-based lesion-symptom mapping. Nat. Neurosci..

[24] Siddiqi S.H., Kording K.P., Parvizi J., Fox M.D. (2022). Causal mapping of human brain function. Nat. Rev. Neurosci..

[25] Fox M.D. (2018). Mapping Symptoms to Brain Networks with the Human Connectome. N. Engl. J. Med..

[26] Schaper F.L.W.V.J., Nordberg J., Cohen A.L., Lin C., Hsu J., Horn A., Ferguson M.A., Siddiqi S.H., Drew W., Soussand L. (2023). Mapping Lesion-Related Epilepsy to a Human Brain Network. JAMA Neurol..

[27] Bowren M., Bruss J., Manzel K., Edwards D., Liu C., Corbetta M., Tranel D., Boes A.D. (2022). Post-stroke outcomes predicted from multivariate lesion-behaviour and lesion network mapping. Brain.

[28] Mandal A.S., Romero-Garcia R., Hart M.G., Suckling J. (2020). Genetic, cellular, and connectomic characterization of the brain regions commonly plagued by glioma. Brain.

[29] Bao H., Ren P., Liang X., Lai J., Bai Y., Liu Y., Lv Z., Hu J., Yan Z., Wang Z. (2024). The Spatial Distribution of Brain Metastasis Is Determined by the Heterogeneity of the Brain Microenvironment. Hum. Brain Mapp..

[30] Liu X., Zhang Q., Li J., Xu Q., Zhuo Z., Li J., Zhou X., Lu M., Zhou Q., Pan H. (2023). Coordinatized lesion location analysis empowering ROI-based radiomics diagnosis on brain gliomas. Eur. Radiol..

[31] Steed T.C., Treiber J.M., Taha B., Engin H.B., Carter H., Patel K.S., Dale A.M., Carter B.S., Chen C.C. (2020). Glioblastomas located in proximity to the subventricular zone (SVZ) exhibited enrichment of gene expression profiles associated with the cancer stem cell state. J. Neuro Oncol..

[32] Wei Y., Li C., Cui Z., Mayrand R.C., Zou J., Wong A.L.K.C., Sinha R., Matys T., Schönlieb C.B., Price S.J. (2023). Structural connectome quantifies tumour invasion and predicts survival in glioblastoma patients. Brain.

[33] Ferreri A.J.M., Illerhaus G., Doorduijn J.K., Auer D.P., Bromberg J.E.C., Calimeri T., Cwynarski K., Fox C.P., Hoang-Xuan K., Malaise D. (2024). Primary central nervous system lymphomas: EHA-ESMO Clinical Practice Guideline for diagnosis, treatment and follow-up. HemaSphere.

[34] Pfau S.J., Langen U.H., Fisher T.M., Prakash I., Nagpurwala F., Lozoya R.A., Lee W.C.A., Wu Z., Gu C. (2024). Characteristics of blood-brain barrier heterogeneity between brain regions revealed by profiling vascular and perivascular cells. Nat. Neurosci..

[35] Wälchli T., Ghobrial M., Schwab M., Takada S., Zhong H., Suntharalingham S., Vetiska S., Gonzalez D.R., Wu R., Rehrauer H. (2024). Single-cell atlas of the human brain vasculature across development, adulthood and disease. Nature.

[36] Alonso M., Petit A.C., Lledo P.M. (2024). The impact of adult neurogenesis on affective functions: of mice and men. Mol. Psychiatr..

[37] Pastor-Alonso O., Syeda Zahra A., Kaske B., García-Moreno F., Tetzlaff F., Bockelmann E., Grunwald V., Martín-Suárez S., Riecken K., Witte O.W. (2024). Generation of adult hippocampal neural stem cells occurs in the early postnatal dentate gyrus and depends on cyclin D2. EMBO J..

[38] Romero-Garcia R., Mandal A.S., Bethlehem R.A.I., Crespo-Facorro B., Hart M.G., Suckling J. (2023). Transcriptomic and connectomic correlates of differential spatial patterning among gliomas. Brain.

[39] Raghu A.L.B., Chen J.A., Valdes P.A., Essayed W.I., Claus E., Arnaout O., Smith T.R., Chiocca E.A., Peruzzi P.P., Bernstock J.D. (2022). Cerebellar High-Grade Glioma: A Translationally Oriented Review of the Literature. Cancers (Basel).

[40] Barisano G., Lynch K.M., Sibilia F., Lan H., Shih N.C., Sepehrband F., Choupan J. (2022). Imaging perivascular space structure and function using brain MRI. Neuroimage.

[41] Ren P., Bao H., Wang S., Wang Y., Bai Y., Lai J., Yi L., Liu Q., Li W., Zhang X. (2024). Multi-scale brain attributes contribute to the distribution of diffuse glioma subtypes. Int. J. Cancer.

[42] Crossley N.A., Mechelli A., Scott J., Carletti F., Fox P.T., McGuire P., Bullmore E.T. (2014). The hubs of the human connectome are generally implicated in the anatomy of brain disorders. Brain.

[43] Peng X., Yang S., Zhou L., Mei Y., Shi L., Zhang R., Shan F., Liu L. (2022). Repeatability and Reproducibility of Computed Tomography Radiomics for Pulmonary Nodules: A Multicenter Phantom Study. Investig. Radiol..

[44] Wong J., Baine M., Wisnoskie S., Bennion N., Zheng D., Yu L., Dalal V., Hollingsworth M.A., Lin C., Zheng D. (2021). Effects of interobserver and interdisciplinary segmentation variabilities on CT-based radiomics for pancreatic cancer. Sci. Rep..

[45] Chen H., Gomez C., Huang C.M., Unberath M. (2022). Explainable medical imaging AI needs human-centered design: guidelines and evidence from a systematic review. npj Digit. Med..

[46] Markello R.D., Arnatkeviciute A., Poline J.B., Fulcher B.D., Fornito A., Misic B. (2021). Standardizing workflows in imaging transcriptomics with the abagen toolbox. eLife.

[47] Vandereyken K., Sifrim A., Thienpont B., Voet T. (2023). Methods and applications for single-cell and spatial multi-omics. Nat. Rev. Genet..

[48] Bressan D., Battistoni G., Hannon G.J. (2023). The dawn of spatial omics. Science.

[49] Greenwald A.C., Darnell N.G., Hoefflin R., Simkin D., Mount C.W., Gonzalez Castro L.N., Harnik Y., Dumont S., Hirsch D., Nomura M. (2024). Integrative spatial analysis reveals a multi-layered organization of glioblastoma. Cell.

[50] Yushkevich P.A., Piven J., Hazlett H.C., Smith R.G., Ho S., Gee J.C., Gerig G. (2006). User-guided 3D active contour segmentation of anatomical structures: significantly improved efficiency and reliability. Neuroimage.

[51] Wu D., Ma T., Ceritoglu C., Li Y., Chotiyanonta J., Hou Z., Hsu J., Xu X., Brown T., Miller M.I., Mori S. (2016). Resource atlases for multi-atlas brain segmentations with multiple ontology levels based on T1-weighted MRI. Neuroimage.

[52] van Griethuysen J.J.M., Fedorov A., Parmar C., Hosny A., Aucoin N., Narayan V., Beets-Tan R.G.H., Fillion-Robin J.C., Pieper S., Aerts H.J.W.L. (2017). Computational Radiomics System to Decode the Radiographic Phenotype. Cancer Res..

[53] Pomponio R., Erus G., Habes M., Doshi J., Srinivasan D., Mamourian E., Bashyam V., Nasrallah I.M., Satterthwaite T.D., Fan Y. (2020). Harmonization of large MRI datasets for the analysis of brain imaging patterns throughout the lifespan. Neuroimage.

[54] Arnatkeviciute A., Fulcher B.D., Fornito A. (2019). A practical guide to linking brain-wide gene expression and neuroimaging data. Neuroimage.

[55] Wagner H.H., Dray S. (2015). Generating spatially constrained null models for irregularly spaced data using Moran spectral randomization methods. Methods Ecol. Evol..

[56] Alfaro-Almagro F., Jenkinson M., Bangerter N.K., Andersson J.L.R., Griffanti L., Douaud G., Sotiropoulos S.N., Jbabdi S., Hernandez-Fernandez M., Vallee E. (2018). Image processing and Quality Control for the first 10,000 brain imaging datasets from UK Biobank. Neuroimage.

[57] Yan C.G., Wang X.D., Zuo X.N., Zang Y.F. (2016). DPABI: Data Processing & Analysis for (Resting-State) Brain Imaging. Neuroinformatics.

[58] Rubinov M., Sporns O. (2010). Complex network measures of brain connectivity: uses and interpretations. Neuroimage.

