# Supplementary material for: Spatial pattern-driven interpretable model and biological correlates in brain glioblastoma-lymphoma differentiation
Source: iScience. 2026 Aug 21;29(9):117281. doi: 10.1016/j.isci.2026.117281 (PMC13524760; doi:10.1016/j.isci.2026.117281)
Supplement: Supplementary file 1 [file mmc1.pdf]

iScience, Volume *29*

Supplemental information

Spatial pattern-driven interpretable model and biological correlates in brain glioblastoma-lymphoma differentiation

Yi Wang, Haohui Chen, Kaiyan Su, Jianrui Li, Tianliang Zhan, Lingyu Kong, Qiang Xu, Gaoping Liu, Xiaorui Su, Wei You, Jianguo Xia, Ji Zhang, Weizhong Tian, Wei Liao, Qiang Yue, Bing Zhang, Weihua Liao, Wei Xing, Dante Mantini, Guangming Lu, Zhiqiang Zhang, and Multi-Centre Cooperative Clinical Study Group for "Lesion-Phenotype Brain Mapping in Brain Tumours"

Document S1. Figures S1–S2 and Tables S1–S2 and S4–S15

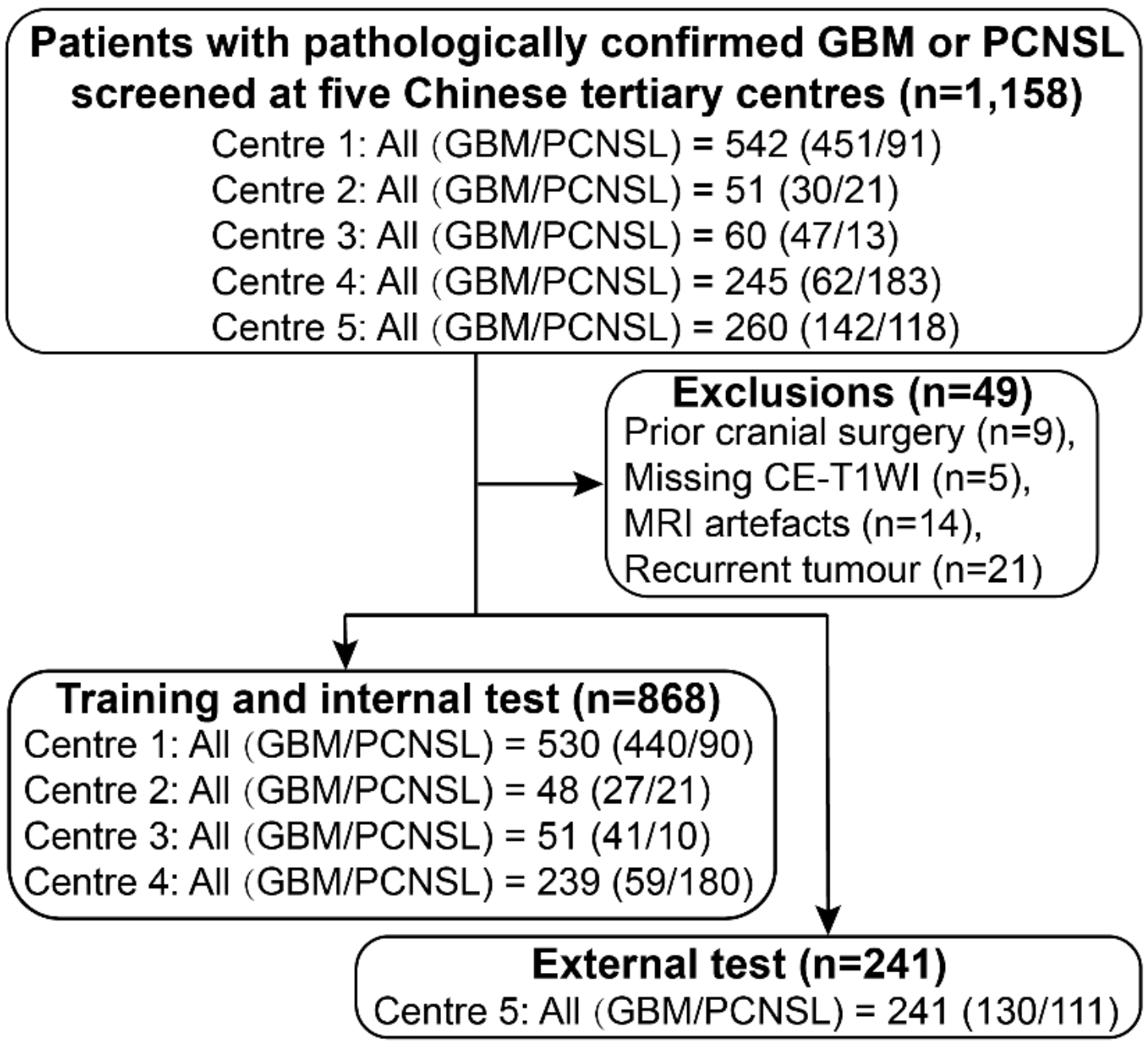

Figure S1. Flow of participants across five tertiary centres, Related to STAR Methods. Overall cohort N = 1,109 (GBM 697; PCNSL 412). Counts are shown as All (GBM/PCNSL). A total of 1,158 pathologically confirmed GBM or PCNSL cases were screened, and 49 were excluded for predefined reasons. Centre 5 was held out as the independent external test set (n = 241), whereas Centres 1–4 formed the development cohort (n = 868), which was randomly split 7:3 into the training set (n = 607) and internal test set (n = 261), stratified by centre and diagnosis. Abbreviations: GBM = glioblastoma; PCNSL = primary central nervous system lymphoma; T1WI-CE = post-contrast T1-weighted MRI.

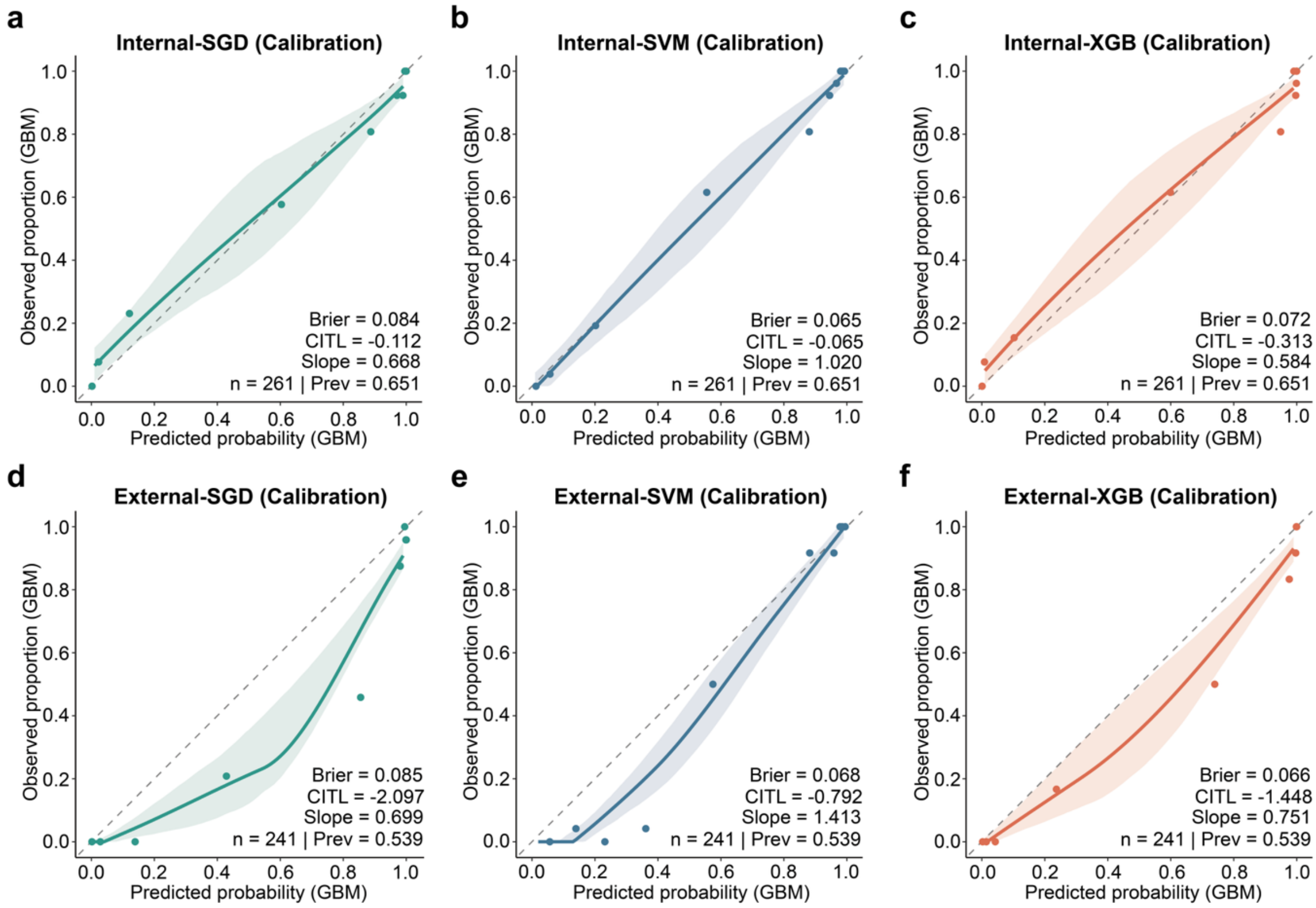

Figure S2. Calibration of spatial radiomics models, Related to Figure 2. (a–c) Internal test cohort (n = 261): **(a)** stochastic gradient descent (SGD), **(b)** support vector machine (SVM), and **(c)** extreme gradient boosting (XGB). **(d–f)** External test cohort from a held-out centre (n = 241): **(d)** SGD, **(e)** SVM, and **(f)** XGB. Calibration curves are plotted for the predicted probability of GBM. The y-axis shows the observed proportion of GBM, and the x-axis shows the predicted probability of GBM. The dashed 45° line indicates perfect calibration; the solid curve is a locally estimated scatterplot smoothing (LOESS) calibration curve with a 95% bootstrap band; dots denote decile-wise observed event rates. Insets report the Brier score, calibration-in-the-large (CITL, ideal = 0), calibration slope (ideal = 1), sample size (n), and prevalence. Calibration metrics are summarised in Table S5.

Table S1. MRI acquisition parameters for T1WI-CE across five centres, Related to STAR Methods

Centre	Vendor/scanner	Protocol name	Field strength (T)	TR (ms)	TE (ms)	Matrix size	Slice thickness (mm)
Centre 1	Siemens	GRE	3.0	280.0	2.5	320×260	5.5
	GE	FSE	1.5	2113.6	25.2	512×512	5.0
	GE	Propeller TSE	3.0	2857.3	26.2	512×512	5.0
Centre 2	Siemens	GRE	3.0	1630.0	2.3	491×491	5.0
	Toshiba	TSE	3.0	678.6	17.0	320×296	6.0
Centre 3	GE	FSE	3.0	719.0	14.5	512×512	5.0
	Siemens	GRE	3.0	1630.0	2.3	512×512	6.0
Centre 4	Philips	SE	1.5	531.7	17.0	256×256	6.0
	GE	FSE	3.0	1765.6	22.0	512×512	5.0
Centre 5	Philips	FFE	1.5	112.3	1.8	640×640	6.0
	United Imaging (UIH)	GRE	3.0	250.0	3.0	512×445	6.0

Note: Abbreviations: T1WI-CE = post-contrast T1-weighted imaging; TR = repetition time; TE = echo time.

Table S2. Significant voxel-wise clusters in the GBM–PCNSL comparison, Related to Figure 1

Brain regions	Peak point (x, y, z)	Q (signed $-\log_{10}q$)	Number of voxels
Posterior superior temporal gyrus, left	−40, −28, 9	−42.98	290,792
Superior temporal gyrus, right	41, −7, −7	−39.47	404,910
Middle frontal gyrus (dorsal prefrontal cortex), left	−28, 41, 35	−7.17	450
Precentral gyrus, left	−46, −7, 47	−6.57	350
Lateral fronto-orbital gyrus, left	−31, 41, −13	−5.32	179
Cerebral peduncle, left	−10, −16, −8	25.25	6,039
Hypothalamus, right	1, −8, −8	18.44	1,541
Splenium of corpus callosum, right	5, −40, 16	13.77	1,736
Cerebellum, left	−10, −49, −37	13.63	22,428
Splenium of corpus callosum, left	−1, −46, 8	12.50	850
Cerebellum, right	1, −52, −28	11.92	12,564
Superior frontal gyrus (posterior segment), left	−13, −16, 71	5.09	249

Note: Clusters were defined in MNI space after voxel-wise FDR control ($q < 0.05$; $|Q| \geq 1.301$), using 3D connected-component analysis with a 26-voxel neighbourhood (faces/edges/corners adjacency) and a minimum cluster extent of 100 voxels. Q = sign (p_PCNSL − p_GBM) × ($-\log_{10}q$); positive Q indicates PCNSL > GBM and negative Q indicates GBM > PCNSL. Region labels follow the JHU-189 atlas at the peak voxel; peak points are reported as MNI RAS+ coordinates (mm).

Table S4. Pairwise AUC comparisons (DeLong test), Related to Figure 2

Algorithm	Dataset	Comparator	AUC (Spatial radiomics)	AUC (Comparator)	ΔAUC	p (DeLong)
SGD	External test	Radiomics	0.9663	0.8968	0.0695	2.12×10^{-4}
		Spatiomics	0.9663	0.7662	0.2001	4.67×10^{-10}
	Internal test	Radiomics	0.9557	0.8869	0.0688	9.23×10^{-7}
		Spatiomics	0.9557	0.7887	0.1670	1.64×10^{-9}
XGB	External test	Radiomics	0.9802	0.8720	0.1082	6.37×10^{-7}
		Spatiomics	0.9802	0.7626	0.2176	8.20×10^{-13}
	Internal test	Radiomics	0.9614	0.9275	0.0339	3.23×10^{-2}
		Spatiomics	0.9614	0.8007	0.1607	1.10×10^{-8}
SVM	External test	Radiomics	0.9786	0.8880	0.0906	4.37×10^{-6}
		Spatiomics	0.9786	0.7745	0.2041	2.70×10^{-11}
	Internal test	Radiomics	0.9668	0.9025	0.0643	3.74×10^{-5}
		Spatiomics	0.9668	0.7736	0.1932	4.76×10^{-10}

Note: Reported p values are unadjusted two-sided DeLong p values from paired ROC comparisons. Multiplicity was addressed using Benjamini–Hochberg FDR correction across the 12 pairwise contrasts; all comparisons remained significant after correction (maximum q = 0.0323). Abbreviations: SGD = stochastic gradient descent; XGB = extreme gradient boosting; SVM = support vector machine; AUC = area under the ROC curve; FDR = false discovery rate.

Table S5. Calibration metrics for the spatial radiomics models in the internal and external test sets, Related to Figure 2

Dataset	Algorithm	Brier	Calibration intercept (CITL)	Calibration slope
Internal test	SGD	0.084	−0.112	0.668
	SVM	0.065	−0.065	1.020
	XGB	0.072	−0.313	0.584
External test	SGD	0.085	−2.097	0.699
	SVM	0.068	−0.792	1.413
	XGB	0.066	−1.448	0.751

Note: Calibration metrics correspond to the predicted probability of GBM, consistent with the calibration curves shown in Figure S2. CITL denotes calibration-in-the-large (ideal = 0); slope ideal = 1. Negative CITL indicates systematic overprediction of GBM probability; slope < 1 indicates over-confident probabilities; slope > 1 indicates under-dispersed probabilities.

Table S6. Threshold-based operating characteristics of the three spatial radiomics classifiers at clinical action thresholds (p_t = 0.25 and p_t = 0.75) in the internal and external test sets, Related to Figure 2

Dataset	Algorithm	Threshold	Sens	Spec	PPV	NPV	Net Benefit
Internal test	SGD	0.25	0.953	0.769	0.885	0.897	0.594
		0.75	0.876	0.857	0.920	0.788	0.421
	SVM	0.25	0.971	0.736	0.873	0.931	0.602
		0.75	0.900	0.912	0.950	0.830	0.494
	XGB	0.25	0.965	0.813	0.906	0.925	0.607
		0.75	0.912	0.879	0.934	0.842	0.467
External test	SGD	0.25	1.000	0.676	0.783	1.000	0.490
		0.75	0.962	0.874	0.899	0.951	0.344
	SVM	0.25	0.992	0.595	0.741	0.985	0.473
		0.75	0.908	0.928	0.937	0.896	0.390
	XGB	0.25	0.992	0.775	0.838	0.989	0.501
		0.75	0.946	0.910	0.925	0.935	0.386

Note: Reported metrics: sensitivity, specificity, positive predictive value (PPV), negative predictive value (NPV), and net benefit. Thresholds (p_t = 0.25/0.75) were pre-specified as liberal and conservative clinical action points to accompany the decision curve analysis (Figure 2c). Net benefit was computed under an opt-in strategy. GBM was treated as the positive class for these DCA-related threshold analyses; therefore, threshold probabilities refer to the predicted probability of GBM. The pre-specified high-sensitivity GBM operating point at τ = 0.30 is reported separately in Tables S8–S9.

Table S7. Confusion matrices of spatial radiomics models at the benchmarking probability threshold of 0.50, Related to Figure 2

Dataset	Algorithm	Actual GBM, n	Actual PCNSL, n	TP: GBM predicted as GBM	FN: GBM predicted as PCNSL	FP: PCNSL predicted as GBM	TN: PCNSL predicted as PCNSL
Internal test	SGD	170	91	157	13	18	73
Internal test	SVM	170	91	160	10	11	80
Internal test	XGB	170	91	159	11	13	78
External test	SGD	130	111	127	3	25	86
External test	SVM	130	111	124	6	10	101
External test	XGB	130	111	125	5	17	94

Note: Abbreviations: GBM = glioblastoma; PCNSL = primary central nervous system lymphoma; SGD = stochastic gradient descent; SVM = support vector machine; XGB = extreme gradient boosting; TP = true positive; FN = false negative; FP = false positive; TN = true negative. At the benchmarking probability threshold of 0.50, GBM was defined as the positive class and PCNSL as the negative class. Accordingly, TP denotes GBM correctly predicted as GBM, FN denotes GBM predicted as PCNSL, FP denotes PCNSL predicted as GBM, and TN denotes PCNSL correctly predicted as PCNSL. Sensitivity, PPV, and F1 in Table 2 refer to GBM classification, whereas specificity and NPV refer to PCNSL as the negative class.

Table S8. Confusion matrices for the three XGB feature-set models at the pre-specified probability threshold (τ = 0.30; positive class = GBM) in the internal and external test sets, Related to Figure 2

Model	Dataset	Reference standard	Index test +	Index test −	Total
Spatial radiomics	Internal test (n = 261)	GBM (positive)	163	7	170
		PCNSL (negative)	16	75	91
	External test (n = 241)	GBM (positive)	128	2	130
		PCNSL (negative)	23	88	111
Radiomics	Internal test (n = 261)	GBM (positive)	162	8	170
		PCNSL (negative)	40	51	91
	External test (n = 241)	GBM (positive)	129	1	130
		PCNSL (negative)	79	32	111
Spatiomics	Internal test (n = 261)	GBM (positive)	167	3	170
		PCNSL (negative)	77	14	91
	External test (n = 241)	GBM (positive)	128	2	130
		PCNSL (negative)	107	4	111

Note: Matrix layout: rows = reference standard (ground truth), columns = index test result. "Index test +" denotes predicted GBM, and "Index test −" denotes predicted PCNSL. Cells correspond to [TP, FN] in the GBM-positive row and [FP, TN] in the PCNSL-negative row. τ = 0.30 was chosen a priori to prioritise sensitivity and was applied unchanged to both test sets. Class encoding: GBM was coded as 1 and PCNSL as 0. Sample sizes: internal test n = 261; external test n = 241. Abbreviations: GBM = glioblastoma; PCNSL = primary central nervous system lymphoma.

Table S9. Threshold-based diagnostic metrics for the three XGB feature-set models at τ = 0.30 (positive class = GBM), with 95% confidence intervals (Wilson) and area under the ROC curve (AUC) in the internal and external test sets, Related to Figure 2

Model	Dataset	Sensitivity	95% CI	Specificity	95% CI	PPV	95% CI	NPV	95% CI	AUC
Spatial radiomics	Internal test	0.959	0.92–0.98	0.824	0.73–0.89	0.911	0.86–0.94	0.915	0.83–0.96	0.961
	External test	0.985	0.95–1.00	0.793	0.71–0.86	0.848	0.78–0.90	0.978	0.92–0.99	0.980
Radiomics	Internal test	0.953	0.91–0.98	0.560	0.46–0.66	0.802	0.74–0.85	0.864	0.75–0.93	0.928
	External test	0.992	0.96–1.00	0.288	0.21–0.38	0.620	0.55–0.68	0.970	0.85–0.99	0.872
Spatiomics	Internal test	0.982	0.95–0.99	0.154	0.09–0.24	0.684	0.62–0.74	0.824	0.59–0.94	0.801
	External test	0.985	0.95–1.00	0.036	0.01–0.09	0.545	0.48–0.61	0.667	0.30–0.90	0.763

Note: Reported threshold-based metrics are sensitivity, specificity, positive predictive value (PPV), and negative predictive value (NPV), each with 95% confidence intervals (Wilson) at τ = 0.30 with GBM as the positive class, plus the threshold-free area under the receiver operating characteristic (ROC) curve (AUC). Confidence intervals were computed from the counts in Table S8. AUC was computed from continuous probabilities rather than thresholded predictions. Benchmarking metrics at the default/validation operating point are summarised in Table 2 of the main text and Table S10; τ = 0.30 results are provided here to satisfy STARD/TRIPOD reporting requirements. Abbreviations: GBM = glioblastoma; PCNSL = primary central nervous system lymphoma; AUC = area under the ROC curve; PPV = positive predictive value; NPV = negative predictive value.

Table S10. Predictive performance across algorithms and model types, Related to Figure 2

Algorithm	Model	Internal AUC	External AUC	ΔAUC	Internal ACC	External ACC	ΔACC
SGD	Radiomics	0.887	0.897	+0.010	0.839	0.830	−0.009
	Spatiomics	0.789	0.766	−0.023	0.762	0.693	−0.069
	Spatial radiomics	0.956	0.966	+0.010	0.881	0.884	+0.003
SVM	Radiomics	0.902	0.888	−0.014	0.820	0.739	−0.081
	Spatiomics	0.774	0.774	+0.000	0.751	0.685	−0.066
	Spatial radiomics	0.967	0.979	+0.012	0.920	0.934	+0.014
XGB	Radiomics	0.928	0.872	−0.056	0.828	0.714	−0.114
	Spatiomics	0.801	0.763	−0.038	0.724	0.622	−0.102
	Spatial radiomics	0.961	0.980	+0.019	0.908	0.909	+0.001

Note: ΔAUC/ΔACC = External − Internal (positive indicates better external generalisation). Abbreviations: SGD = stochastic gradient descent; SVM = support vector machine; XGB = extreme gradient boosting; AUC = area under the ROC curve; ACC = accuracy.

Table S11. Centre-stratified performance and calibration of spatial radiomics models, Related to Figure 2

Centre	Dataset	Algorithm	n	GBM	PCNSL	PCNSL prevalence	AUC	95% CI	ACC	SENS	SPEC	PPV	NPV	F1	Brier	CITL	Calibration slope
1	Internal	SGD	164	134	30	0.183	0.958	0.930–0.986	0.915	0.940	0.800	0.955	0.750	0.947	0.064	0.790	0.757
1	Internal	SVM	164	134	30	0.183	0.954	0.924–0.984	0.921	0.940	0.833	0.962	0.758	0.951	0.061	0.584	0.924
1	Internal	XGB	164	134	30	0.183	0.944	0.911–0.978	0.921	0.948	0.800	0.955	0.774	0.951	0.064	0.380	0.541
2	Internal	SGD	14	8	6	0.429	0.958	NE	0.786	1.000	0.500	0.727	1.000	0.842	0.176	NE	NE
2	Internal	SVM	14	8	6	0.429	1.000	NE	1.000	1.000	1.000	1.000	1.000	1.000	0.024	NE	NE
2	Internal	XGB	14	8	6	0.429	1.000	NE	1.000	1.000	1.000	1.000	1.000	1.000	0.006	NE	NE
3	Internal	SGD	18	17	1	0.056	1.000	NE	0.833	0.824	1.000	1.000	0.250	0.903	0.121	NE	NE
3	Internal	SVM	18	17	1	0.056	1.000	NE	0.889	0.882	1.000	1.000	0.333	0.938	0.082	NE	NE
3	Internal	XGB	18	17	1	0.056	1.000	NE	0.778	0.765	1.000	1.000	0.200	0.867	0.127	NE	NE
4	Internal	SGD	65	11	54	0.831	0.960	0.877–1.000	0.831	0.818	0.833	0.500	0.957	0.621	0.105	-2.359	0.791
4	Internal	SVM	65	11	54	0.831	0.981	0.925–1.000	0.908	1.000	0.889	0.647	1.000	0.786	0.080	-1.897	1.536
4	Internal	XGB	65	11	54	0.831	0.975	0.909–1.000	0.892	1.000	0.870	0.611	1.000	0.759	0.092	-3.320	0.721
5	External	SGD	241	130	111	0.461	0.966	0.944–0.989	0.884	0.977	0.775	0.836	0.966	0.901	0.085	-2.097	0.699
5	External	SVM	241	130	111	0.461	0.979	0.961–0.997	0.934	0.954	0.910	0.925	0.944	0.939	0.068	-0.792	1.413
5	External	XGB	241	130	111	0.461	0.980	0.963–0.997	0.909	0.962	0.847	0.880	0.949	0.919	0.066	-1.448	0.751

Note: Centres 1–4 were evaluated only in their held-out internal-test subsets; Centre 5 was the held-out external test centre. PCNSL prevalence denotes the proportion of PCNSL cases within each centre-specific test subset and is shown for cohort composition. GBM was coded as label 1 and therefore the positive class for SENS, PPV, F1, CITL, and calibration slope; SPEC and NPV refer to PCNSL as the negative class. Calibration metrics correspond to the predicted probability of GBM. NE = not estimated because the centre-specific test subset was too small or markedly imbalanced. Abbreviations: SENS = sensitivity; SPEC = specificity; PPV = positive predictive value; NPV = negative predictive value; CITL = calibration-in-the-large; NE = not estimated.

Table S12. Centre-balanced sensitivity analysis of spatial radiomics models, Related to Figure 2

Algorithm	Macro-average AUC	AUC SD	Centre AUC range	Macro ACC	Macro Brier	Pooled internal AUC	Pooled external AUC
SGD	0.968	0.018	0.958–1.000	0.850	0.110	0.956	0.966
SVM	0.983	0.019	0.954–1.000	0.930	0.063	0.967	0.979
XGB	0.980	0.023	0.944–1.000	0.900	0.071	0.961	0.980

Note: Abbreviations: AUC = area under the ROC curve; ACC = accuracy; SD = standard deviation. Macro-average metrics assign equal weight to each of the five centres, irrespective of centre sample size. Centre-specific estimates from Centres 2 and 3 should be interpreted cautiously because their internal-test subsets were small, and Centre 3 included only one PCNSL case. Full centre-specific cohort composition and allocation are provided in Figure S1. Macro-average AUC, ACC, and Brier score were calculated as the unweighted arithmetic means of the corresponding centre-specific estimates; pooled internal and external estimates are shown for reference only.

Table S13. Interobserver and registration-perturbation robustness of atlas-anchored spatial features, Related to STAR Methods

Analysis	Metric	Result
Native-space interobserver segmentation	Dice, median (IQR)	0.915 (0.862–0.942)
Native-space interobserver segmentation	Tumour-volume ICC	0.967
MNI-normalised interobserver segmentation	Dice, median (IQR)	0.889 (0.786–0.931)
MNI-normalised interobserver segmentation	Tumour-volume ICC	0.964
Atlas-anchored spatial descriptors, interobserver	Feature ICC, median (IQR)	0.966 (0.911–0.986)
Atlas-anchored spatial descriptors, interobserver	Features with ICC ≥ 0.75	89.7%
Atlas-anchored spatial descriptors, interobserver	Features with ICC ≥ 0.90	77.7%
Atlas-anchored spatial descriptors, interobserver	Total spatial-descriptor score ICC	0.942
Atlas-anchored spatial descriptors, interobserver	Per-case cosine similarity, median	0.988
Atlas-anchored spatial descriptors, interobserver	Binary parcel-involvement agreement, median	0.979
±2 mm registration perturbation	Feature ICC, median (IQR)	0.989 (0.968–0.995)
±2 mm registration perturbation	Features with ICC ≥ 0.75	95.7%
±2 mm registration perturbation	Features with ICC ≥ 0.90	92.4%
±2 mm registration perturbation	Total spatial-descriptor score ICC	0.997
±2 mm registration perturbation	Per-case cosine similarity, median	0.990
±2 mm registration perturbation	Binary parcel-involvement agreement, median	0.989

Note: Interobserver robustness was assessed in 50 paired cases independently segmented by two readers. Atlas-anchored spatial descriptors refer to the same JHU-189 atlas-based spatial features used in the main modelling pipeline. Registration perturbation analysis was performed by shifting the Reader 1 MNI-normalised masks by ±2 mm along each of the three image axes, followed by re-extraction of the same atlas-anchored spatial descriptors. Pooled perturbation results were calculated across 300 comparisons, corresponding to 50 cases × 6 perturbation directions. Abbreviations: ICC = intraclass correlation coefficient; IQR = interquartile range; MNI = Montreal Neurological Institute.

Table S14. Hyperparameter search grids and selected configurations for each algorithm and feature specification, Related to STAR Methods

Algorithm	Model	Grid (key parameters)	Selected settings
SGD	Radiomics	penalty ∈ {L2, elasticnet}; α ∈ {1e-4, 1e-3, 1e-2}; l1_ratio ∈ {0, 0.15, 0.30}; learning_rate ∈ {optimal, adaptive}	elasticnet; α = 0.01; l1_ratio = 0.15; learning_rate = optimal; max_iter = 1000
	Spatiomics	penalty ∈ {L2, elasticnet}; α ∈ {1e-4, 1e-3, 1e-2}; l1_ratio ∈ {0, 0.15, 0.30}; learning_rate ∈ {optimal, adaptive}; class_weight ∈ {None, balanced}	elasticnet; α = 0.01; l1_ratio = 0.30; learning_rate = optimal; max_iter = 2000
	Spatial radiomics	penalty ∈ {L2, elasticnet}; α ∈ {1e-4, 1e-3, 1e-2}; l1_ratio ∈ {0, 0.15, 0.30}; learning_rate ∈ {optimal, adaptive}; η_0 ∈ {0.0, 0.01}	elasticnet; α = 0.01; l1_ratio = 0.30; learning_rate = adaptive; η_0 = 0.01; max_iter = 1000
SVM	Radiomics	C ∈ {0.1, 1, 10}; gamma ∈ {scale, 0.01, 0.1}; class_weight ∈ {None, balanced}	C = 10; gamma = 0.01; class_weight = None
	Spatiomics	C ∈ {0.1, 1, 10}; gamma ∈ {scale, 0.01, 0.1}; class_weight ∈ {None, balanced}	C = 1; gamma = scale; class_weight = balanced
	Spatial radiomics	C ∈ {0.1, 1, 10}; gamma ∈ {scale, 0.005, 0.05}; class_weight ∈ {None, balanced}	C = 1; gamma = 0.005; class_weight = None
XGB	Radiomics	n_estimators ∈ {300, 500}; max_depth ∈ {3, 4}; learning_rate ∈ {0.03, 0.1}; subsample ∈ {0.7, 1.0}; colsample_bytree ∈ {0.6, 0.8, 1.0}; reg_lambda ∈ {1.0, 10.0}; min_child_weight ∈ {1, 3}	n_estimators = 300; max_depth = 4; learning_rate = 0.1; subsample = 0.7; colsample_bytree = 0.8; reg_lambda = 10.0; min_child_weight = 3
	Spatiomics	n_estimators ∈ {300, 500}; max_depth ∈ {2, 3, 4}; learning_rate ∈ {0.03, 0.1}; subsample ∈ {0.8, 1.0}; colsample_bytree ∈ {0.6, 0.8, 1.0}; reg_lambda ∈ {1.0, 10.0}; min_child_weight ∈ {1, 3}	n_estimators = 300; max_depth = 3; learning_rate = 0.03; subsample = 1.0; colsample_bytree = 0.8; reg_lambda = 10.0; min_child_weight = 1
	Spatial radiomics	n_estimators ∈ {300, 500}; max_depth ∈ {3, 4, 5}; learning_rate ∈ {0.03, 0.1}; subsample ∈ {0.7, 0.9}; colsample_bytree ∈ {0.7, 1.0}; reg_lambda ∈ {1.0, 10.0}; min_child_weight ∈ {1, 3}	n_estimators = 300; max_depth = 4; learning_rate = 0.03; subsample = 0.9; colsample_bytree = 1.0; reg_lambda = 1.0; min_child_weight = 1

Note: SVM used the RBF kernel. Model selection was based on AUC in stratified five-fold cross-validation within the training partition. NeuroCombat parameters were estimated using training data only; neither held-out test set was used to estimate or update harmonisation parameters. Because the independent external centre was not represented during training, no centre-specific ComBat adjustment was applied to the external radiomics features. Parameters not listed in the grid, including max_iter for SGD, were fixed a priori. Feature selection used SelectKBest (radiomics, k = 10; spatiomics, k = 50). For spatial radiomics, 30 radiomics and 50 spatiomics features were retained and concatenated for subsequent modelling. Abbreviations: SGD = stochastic gradient descent; SVM = support vector machine; XGB = extreme gradient boosting; RBF = radial basis function; AUC = area under the ROC curve.

Table S15. Data-processing, model-development, and held-out evaluation pipeline, Related to STAR Methods

Pipeline step	Before data splitting	Within training / cross-validation	After model selection / final fitting	Held-out internal test	Independent external test (Centre 5)
Case-wise image processing and feature extraction	Segmentation, MNI normalisation, radiomics extraction, and atlas-based spatial feature extraction were performed independently for each patient; no cohort-level parameters or outcomes were used.	Not re-estimated.	No change.	Same case-wise procedures.	Same case-wise procedures.
Dataset partitioning	Centre 5 was designated as the independent external test set; the remaining 868 cases were split into training (n=607) and internal test (n=261), stratified by site and diagnosis.	Only the training partition was used for model development.	Partitions remained fixed.	Held out from model development.	Held out from model development and centre-specific adaptation.
neuroCombat harmonisation	Not estimated before splitting.	Parameters were estimated using only the training partition, with imaging centre as the batch variable and age and sex as covariates. No held-out test data were used to estimate or update parameters.	Training-derived parameters were locked.	No parameter re-estimation; no internal-test data were used to estimate or update harmonisation parameters.	Centre 5 was an unseen batch; no centre-specific or extrapolative ComBat adjustment was applied, and no external data were used for adaptation.

Variance filtering, standardisation, correlation pruning, and feature reduction	Not fitted before splitting.	Fitted using training data only; where cross-validation was used, parameters were learned from each training fold and applied to the corresponding validation fold.	Refitted on the complete training partition using the selected configuration.	Locked transformations applied without refitting.	Locked transformations applied without refitting.
Hyperparameter selection	Not performed.	Selected by stratified five-fold cross-validation using AUC within the training partition.	Selected settings were fixed.	Not used for model selection or tuning.	Not used for model selection or tuning.
Final model fitting	Not performed.	Candidate models were evaluated only within the training partition.	Final SGD, SVM, and XGB pipelines were fitted on the complete training partition.	No model updating.	No model updating.
Operating thresholds	Not applicable.	No threshold optimisation used data from either held-out test set.	The benchmarking threshold of 0.50 and the high-sensitivity GBM operating threshold of 0.30 were fixed before held-out evaluation.	Applied once.	Applied once.
Performance evaluation	Not performed.	Cross-validation was used only for model selection.	The complete pipeline was locked before held-out evaluation.	Evaluated once; no preprocessing, feature-selection, tuning, recalibration, or threshold parameters were estimated from this set.	Evaluated once as direct transportability to an unseen centre without centre-specific adaptation.

Note: All data-driven preprocessing, feature-selection, and model-development steps were restricted to the training data. Neither held-out test set was used to estimate or update neuroCombat, scaling, feature-selection, model, recalibration, or threshold parameters.
